# Caveolin-1 deficiency induces a MEK-ERK1/2-Snail-1-dependent epithelial–mesenchymal transition and fibrosis during peritoneal dialysis

**DOI:** 10.15252/emmm.201404127

**Published:** 2014-12-30

**Authors:** Raffaele Strippoli, Jesús Loureiro, Vanessa Moreno, Ignacio Benedicto, María Luisa Pérez Lozano, Olga Barreiro, Teijo Pellinen, Susana Minguet, Miguel Foronda, Maria Teresa Osteso, Enrique Calvo, Jesús Vázquez, Manuel López Cabrera, Miguel Angel del Pozo

**Affiliations:** 1 Department of Vascular biology and Inflammation, Centro Nacional de Investigaciones CardiovascularesMadrid, Spain; 2 Department of Cellular Biotechnologies and Haematology, Sapienza University of RomeRome, Italy; 3 Molecular Biology Unit, Hospital Universitario de la Princesa, Instituto de Investigación Sanitaria Princesa (IP)Madrid, Spain; 4 CIBERehd, Instituto de Salud Carlos IIIMadrid, Spain

**Keywords:** caveolin-1, epithelial–mesenchymal transition, fibrosis, MEK-ERK1/2 pathway, peritoneal dialysis

## Abstract

Peritoneal dialysis (PD) is a form of renal replacement therapy whose repeated use can alter dialytic function through induction of epithelial–mesenchymal transition (EMT) and fibrosis, eventually leading to PD discontinuation. The peritoneum from Cav1^−/−^ mice showed increased EMT, thickness, and fibrosis. Exposure of Cav1^−/−^ mice to PD fluids further increased peritoneal membrane thickness, altered permeability, and increased the number of FSP-1/cytokeratin-positive cells invading the sub-mesothelial stroma. High-throughput quantitative proteomics revealed increased abundance of collagens, FN, and laminin, as well as proteins related to TGF-β activity in matrices derived from Cav1^−/−^ cells. Lack of Cav1 was associated with hyperactivation of a MEK-ERK1/2-Snail-1 pathway that regulated the Smad2-3/Smad1-5-8 balance. Pharmacological blockade of MEK rescued E-cadherin and ZO-1 inter-cellular junction localization, reduced fibrosis, and restored peritoneal function in Cav1^−/−^ mice. Moreover, treatment of human PD-patient-derived MCs with drugs increasing Cav1 levels, as well as ectopic Cav1 expression, induced re-acquisition of epithelial features. This study demonstrates a pivotal role of Cav1 in the balance of epithelial versus mesenchymal state and suggests targets for the prevention of fibrosis during PD.

## Introduction

Epithelial–mesenchymal transition (EMT) is a complex and stepwise process that occurs during embryonic development and tumor progression, and that has recently been described in chronic inflammatory and fibrogenic diseases (Kalluri & Weinberg, 2009; Thiery *et al*, 2009; Xu *et al*, 2009). EMT is characterized by the disruption of intercellular junctions, replacement of apical–basolateral with front-to-back polarity, and acquisition of migratory and invasive phenotypes. During the establishment of EMT, epithelial cells undergo a complex reprogramming of their cell proteome, in which proteins commonly expressed by epithelia (E-cadherin, cytokeratins) are lost, while cells gain expression of mesenchymal markers (N-cadherin, vimentin, α-smooth muscle actin (α-SMA), fibroblast specific protein-1 (FSP-1)). Moreover, cells acquire the ability to produce extracellular matrix components (ECM) such as fibronectin (FN) and collagen, as well as metalloproteinases and inflammatory, fibrogenic, and angiogenic factors. EMT is triggered by a complex interplay of extracellular signals, including ECM components and soluble growth factors and cytokines, such as members of the transforming growth factor (TGF)-β, fibroblast growth factor (FGF), epidermal growth factor (EGF), and hepatocyte growth factor (HGF) families (Thiery *et al*, 2009; Xu *et al*, 2009; Loureiro *et al*, 2011). The molecular mechanisms controlling the initiation and progression of EMT appear to be multifactorial and cell type specific. The crucial disruption of intercellular junctions between epithelial cells in EMT involves a pivotal action of the transcription factors Snail-1, Zeb, and members of the basic helix-loop-helix (bHLH) family, which repress E-cadherin expression (Peinado *et al*, 2007). The expression of these transcription factors is in turn controlled by a complex network of signaling molecules, including Smads, integrin-linked kinase (ILK), phosphatidylinositol 3-kinase (PI3K), mitogen-activated protein kinases (MAPKs), glycogen synthase kinase (GSK) 3β, and nuclear factor κB (NF-κB) (Zavadil & Bottinger, 2005; Thiery *et al*, 2009).

TGF-β-induced signaling pathways are central to many experimental models of EMT. TGF-β signals through a heterotetrameric complex of two type I and two type II transmembrane serine–threonine kinase receptors. In response to TGF-β, type II receptor kinases phosphorylate type I receptors, leading to activation of cellular responses (Xu *et al*, 2009). Activated type I receptors directly activate Smad2 and Smad3 through C-terminal phosphorylation. Phosphorylated Smad2 and Smad3 then form trimers with Smad4, and translocate to the nucleus, where they associate and cooperate with DNA-binding transcription factors to regulate target gene transcription. In addition to this classical signaling pathway, TGF-β receptors can also elicit signaling responses through important effector pathways more known for their activation by tyrosine kinase receptors, such as MAP kinases, Rho GTPases, and PI3K (Xu *et al*, 2009).

A particular form of EMT occurs in peritoneal mesothelial cells (MCs) subjected to repeated inflammatory stimulus, such as those occurring during peritoneal dialysis (PD). PD is an alternative to hemodialysis for the treatment of end-stage renal disease (Grassmann *et al*, 2005). Currently, PD accounts for more than 10% of all forms of renal replacement therapy worldwide (Grassmann *et al*, 2005). During PD, the peritoneal membrane (PM) acts as a permeable barrier across which ultrafiltration and diffusion take place (Aroeira *et al*, 2007). Continual exposure to hyperosmotic, hyperglycemic and acidic dialysis solutions, as well as episodes of peritonitis and hemoperitoneum, can cause acute and chronic inflammation and injury to the PM, which undergoes progressive fibrosis, angiogenesis, and vasculopathy, eventually leading to discontinuation of PD. Among the wide array of extracellular factors implicated in this process, TGF-β proteins play a major role (Margetts *et al*, 2005; Loureiro *et al*, 2011). The PM undergoes EMT during PD (Yanez-Mo *et al*, 2003), and a variety of molecular mechanisms have been shown to play a role in the regulation of MC plasticity. Signals transduced via TAK1-NF-κB and an ERK1/2-NF-κB-Snail-1 axis control downregulation of E-cadherin and the induction of a mesenchymal state. Conversely, p38 MAPK promotes the maintenance of E-cadherin levels and the epithelial state (Strippoli *et al*, 2008, 2010, 2012).

Here, we investigated the possible role played by caveolin-1 (Cav1) during EMT. Caveolins are the main structural components of caveolae, 60- to 80-nm-diameter flask-shaped plasma membrane invaginations implicated in viral entry, vesicle transport, lipid metabolism, cell signaling, mechanosensing, and mechanotransduction (Chidlow & Sessa, 2010; Hansen & Nichols, 2010; Parton & del Pozo, 2013). Cav1 is widely expressed, and genetic loss of Cav1 in mice results in almost complete loss of caveolae *in vivo*, associated with altered lipid metabolism, pulmonary hypertension and fibrosis, nitric oxide (NO) dysfunction, and cardiac abnormalities (Drab *et al*, 2001; Razani *et al*, 2001a). We recently reported that stromally expressed Cav1 promotes biomechanical remodeling of the tumor microenvironment, thus fostering tumor cell local invasion and distant metastasis [23]. Here, we demonstrate that absence of Cav1 in mice leads to increased EMT and fibrosis in the peritoneal membrane (PM), both in basal conditions and upon exposure to PD fluids. Pharmacological inhibition of the MEK-ERK1/2-Snail-1 pathway, which is hyperactivated in the absence of Cav1, rescued EMT fibrosis in wild-type (WT) and in Cav1^−/−^ mice. Moreover, endothelial cells (EC), which form part of the PM and share a mesodermal origin with MCs, acquired mesenchymal-like features in the absence of Cav1, and overexpression of Cav1 in MCs from patients undergoing PD promoted a reverse EMT (mesenchymal-to-epithelial transition; MET). Our results describe a crucial role of Cav1 in the cellular changes leading to EMT and fibrosis and indicate the therapeutical potential of MEK pharmacological inhibition for the treatment of PM dysfunction during PD.

## Results

### Cav1 absence promotes an EMT phenotype

**Figure 1:**
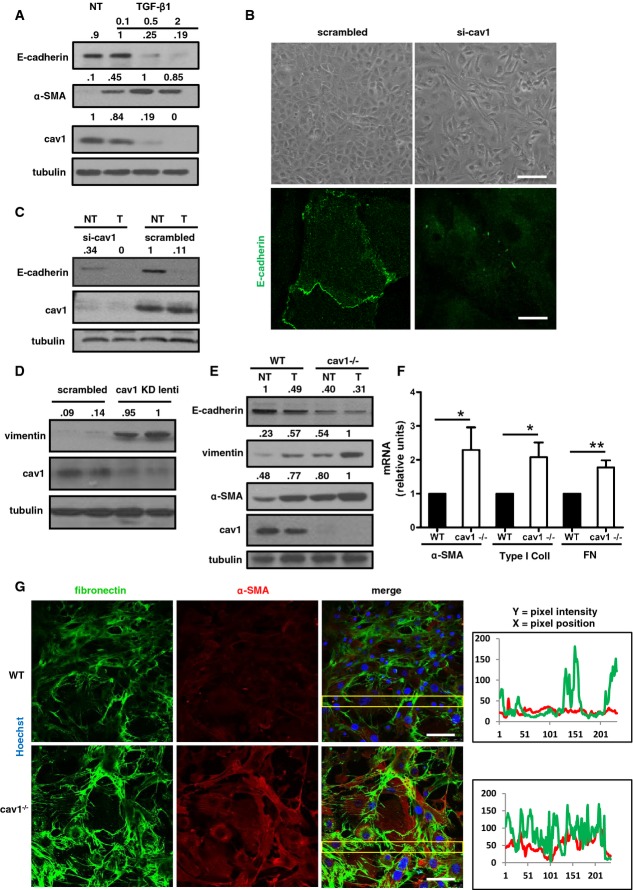
Absence of Cav1 promotes EMT-related changes Western blots (WB) showing the expression of E-cadherin, α-SMA, and Cav1 in total lysates of HPMCs not treated (NT) or treated with different doses of TGF-β1 for 24 h. Tubulin was detected as a loading control.Top: Photomicrographs of confluent monolayers of HPMCs transfected with either control or Cav1-targeting siRNAs. Scale bar: 100 μm. For quantification of elliptical factor, see Supplementary Fig S1A. Bottom: Confocal immunofluorescence (IF) of HPMCs treated as above. Cells were fixed, permeabilized, and stained with a monoclonal antibody against E-cadherin. The IF shown is representative of three independent experiments. Scale bar: 15 μm.WB showing the expression of E-cadherin and Cav1 in whole-cell lysates of HPMCs transfected with control (scrambled) or Cav1-targeting siRNAs and then left untreated (NT) or stimulated for 24 h with TGF-β1 (1 ng/ml; T).Western blots showing the expression of vimentin and Cav1 in whole-cell lysates of HPMCs infected with control or Cav1-targeting shRNA lentivirus.Western blots showing the expression of E-cadherin, vimentin, α-SMA, and Cav1 in whole-cell lysates of MCs from WT and Cav1^−/−^ mice not treated (NT) or treated with 2 ng/ml TGF-β1 for 48 h (T).Differential mRNA expression of α-SMA, type I collagen, and fibronectin (FN) in MCs from WT and Cav1^−/−^ mice. Quantitative RT–PCR was performed on total RNA. Histone H3 mRNA levels were used for normalization. Bars represent means ± SEM of duplicate determinations in four independent experiments. *P =* *0.02, and **P =* *0.0079 for Cav1^−/−^ with respect to WT samples.Confocal IF of MCs from WT (top) and Cav1^−/−^ mice (bottom). Cells were fixed and stained with a polyclonal antibody against fibronectin (green) and a monoclonal antibody against α-SMA (red). Nuclei were stained with Hoechst 33342 (blue). The IF shown is representative of three independent experiments. Scale bar: 50 μm. Data information: In (A, C–E), numbers above panels are ratios of the specific and the tubulin band intensities and data are representative of three independent experiments Source data are available online for this figure.

Acute and chronic inflammatory events linked to PD induce EMT and peritoneal membrane fibrosis (Yanez-Mo *et al*, 2003), in a process largely dependent on TGF-β1 production (Margetts *et al*, 2005). TGF-β1 stimulation markedly reduced Cav1 levels in human primary MCs (HPMCs) in a dose-dependent manner (Fig1A), and this was paralleled by reduced expression of the epithelial marker E-cadherin and induction of the mesenchymal marker α-SMA.

To test the potential involvement of Cav1 in EMT, we first silenced Cav1 expression in HPMCs. Cav1 silencing led to the loss of the typical HPMC cobblestone cell architecture and the acquisition of the characteristic EMT spindle shape (Fig1B, top, for quantification see Supplementary Fig S1A). Moreover, Cav1 knockdown led to reduction of E-cadherin protein expression and also to its disappearance from cell junctions (Fig1B, bottom). E-cadherin downregulation paralleled relevant changes in distribution of proteins expressed in cell junctions, such as α- β- γ-catenin and occludin (Supplementary Fig S1B–E).

**Figure 2:**
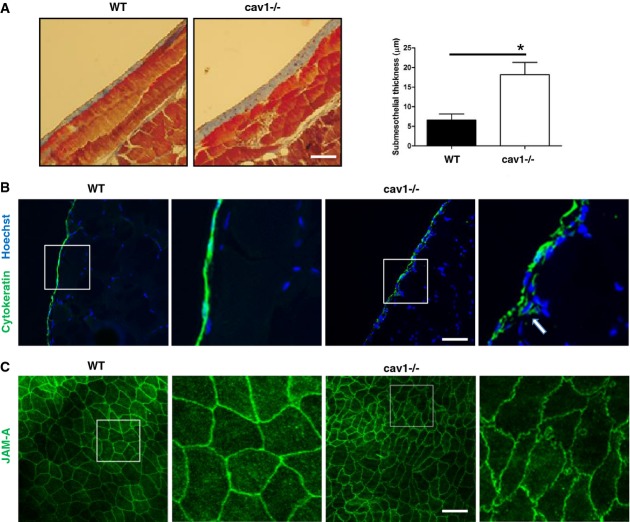
Morphological features of PM in Cav1^−/−^ mice recapitulate ongoing EMT Microscopy analysis of parietal peritoneum from WT (left) and Cav1^−/−^ mice (right). Sections were stained with Masson's trichrome. Magnification: 200×. *P =* *0.016 compared with sections from WT mice. Scale bar: 50 μm.Immunofluorescence (IF) analysis of parietal peritoneal tissue sections from WT (left) and Cav1^−/−^ mice (right). The sections were stained for pan-cytokeratin (green) with Hoechst counterstaining. Scale bar: 50 μm.Confocal IF of parietal peritoneal sections from WT and Cav1^−/−^ mice. The peritoneal membranes were whole-mount-stained with anti-JAM-A monoclonal antibody. Scale bar: 30 μm. Data information: Images are representative of the analysis of five mice per group.

**Figure 3:**
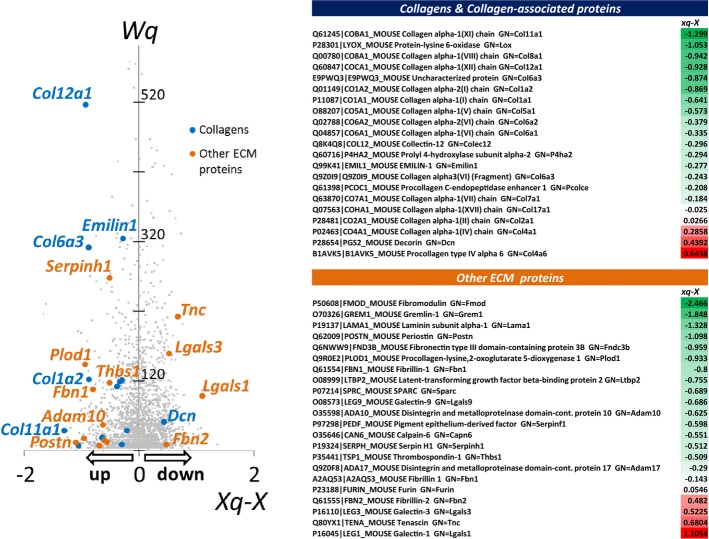
High-throughput quantitative proteomics analysis of proteins from WT and Cav1^−/−^ cells The points represent the distribution of corrected log2 ratios (WT/KO) (*Xq-X*) of protein quantifications according to their statistical weight (*Wq*, a parameter that measures the accuracy of protein quantifications, according to the WSPP model (Navarro *et al*, 2014)). The corrected log2 ratios of collagens and associated proteins, and of other ECM-related proteins, are listed in the right table. Negative values represent an increase in KO cells, and the colors in the table are graded from green (increase) to red (decrease).

On the other hand, the expression of vimentin, a mesenchymal marker, was increased (Fig1D and E). A similar downregulation of epithelial and induction of mesenchymal markers was detected in *ex vivo* cultured MCs from Cav1^−/−^ mice (Fig1E). Transcript analysis of Cav1^−/−^ MCs revealed increased expression relative to wild-type (WT) cells of α-SMA and the ECM proteins FN and type I collagen (Fig1F), and increased expression of FN and α-SMA was confirmed by confocal immunocytochemistry (Fig1G). The parietal peritoneum of Cav1^−/−^ mice showed a significant thickening of the sub-mesothelial space and deposition of ECM (Fig2A). High-throughput quantitative proteomics analysis of extracellular matrices derived from WT and Cav1^−/−^ murine embryonic fibroblasts (MEFs) revealed an increase in abundance of ECM proteins such as collagens, FN, and laminin in matrices from Cav1^−/−^ MEFs (Fig3).

Immunohistochemical analysis showed that the MC monolayer is conserved in the peritoneum of Cav1^−/−^ mice (Fig2B). However, cytokeratin-positive cells (true MCs) in these sections had loose intercellular connections and showed a tendency to invade the sub-mesothelial stroma (see arrows). Whole-mount staining of the peritoneal membrane showed that MCs from Cav1^−/−^ mice have an elongated, spindlelike shape, with punctate staining for JAM-A, a marker of cellular tight junctions (Fig2C).

### MEK-ERK1/2-Snail-1 hyperactivation participates in EMT induction by Cav1^−/−^ MCs

**Figure 4:**
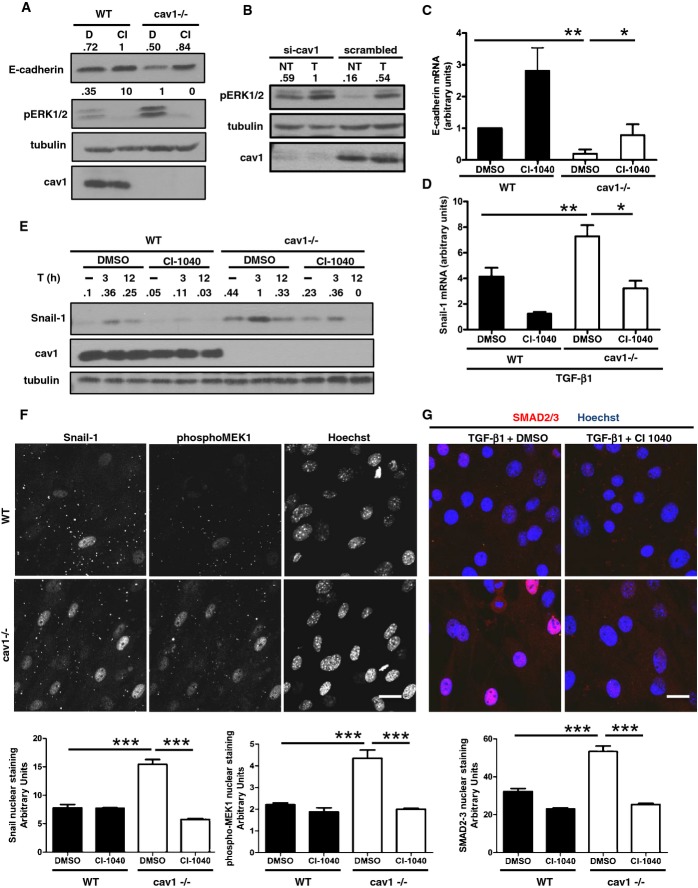
ERK/Snail-1 hyperactivation in Cav1-deficient mouse and human MCs A WB for E-cadherin, phospho-ERK1/2, and Cav1 in total cell lysates of MCs. Cells were treated with vehicle (DMSO, D) or CI-1040 (CI, 2 μM) for 24 h. Tubulin was detected as a loading control. Data are representative of three independent experiments.B WB for phospho-ERK1/2 and Cav1 in total cell lysates of HPMCs transfected with control or Cav1-targeting siRNAs and then stimulated as indicated for 30 min with TGF-β1 (T, 1 ng/ml). Scrambled: cells transfected with control siRNA. Tubulin was detected as a loading control. Data are representative of three independent experiments.C Effect of pharmacologic inhibition of MEK on E-cadherin mRNA expression in MCs from WT or Cav1^−/−^ mice. Cells were treated with 2 μM CI-1040 or DMSO for 24 h, and mRNA was amplified from total RNA by quantitative RT–PCR; histone H3 mRNA expression was used for normalization. **P =* *0.0015; *P =* *0.02.D Effect of pharmacologic inhibition of MEK on Snail-1 mRNA expression in MCs from WT or Cav1^−/−^ mice. Cells were pretreated with 2 μM CI-1040 (CI) or DMSO (D) for 1 h and stimulated with TGF-β1 (2 ng/ml) for 12 h. Normalization as in (C). Bars represent means ± SEM of duplicate determinations in five independent experiments. **P =* *0.0037; *P =* *0.04.E Effect of pharmacologic inhibition of MEK on Snail-1 protein expression in MCs from WT or Cav1^−/−^ mice. Cells were pretreated with 2 μM CI-1040 or DMSO for 1 h and then stimulated as indicated with TGF-β1 (T, 2 ng/ml) for 3 or 12 h. Tubulin was detected as a loading control. Data are representative of three independent experiments.F,G Top: Confocal IF of MCs from WT and Cav1^−/−^ mice. (F) Cells were fixed and immunostained for phospho-MEK1 and Snail-1. Nuclei were stained with Hoechst 33342. Scale bar: 15 μm. (G) Cells were pretreated with 2 μM CI-1040 or DMSO for 1 h and then stimulated with TGF-β1 (2 ng/ml) for 12 h. Cells were fixed and immunostained for SMAD2-3 (red). Nuclei were stained with Hoechst 33342 (blue). Scale bar: 15 μm. Bottom: The histograms show mean fluorescence intensities of nuclear staining quantified using the software LAS-AS from Leica. Bars represent s.e.m. A total of at least 150 cells were analyzed per condition from three different experiments. Data are representative of three independent experiments. ***P <* *0.001 and ***P <* *0.001 for Snail-1(F, left); ***P =* *0.0008 and ***P <* *0.001 for phospho-MEK1 (F, right); and ***P <* *0.001 and ***P <* *0.001 for SMAD2-3 (G) nuclear staining. Source data are available online for this figure.

Cav1 deficiency is linked to increased activity of AKT and ERK1/2 in heart and lung (Wang *et al*, 2006; Murata *et al*, 2007). We found that ERK1/2 is basally hyperactivated in MCs from Cav1^−/−^ mice and in Cav1-silenced HPMCs (Fig4A and B), and basal and TGF-β1-stimulated ERK1/2 activity was strongly inhibited by the specific MEK inhibitor CI-1040 (Sebolt-Leopold *et al*, 1999) (Fig4A). Moreover, CI-1040 increased E-cadherin transcript and protein expression in Cav1^−/−^ MCs (Fig4A and C).

The ERK pathway is a major inducer of Snail-1 family proteins, which directly repress E-cadherin expression (Cano *et al*, 2000; Strippoli *et al*, 2008). Hyperactivation of ERK1/2 in Cav1^−/−^ MCs could thus contribute to the observed low E-cadherin expression by increasing expression of Snail-1 proteins. MCs from Cav1^−/−^ mice express more Snail-1 transcript and protein than WT MCs, both basally and upon TGF-β1 stimulation (Fig4D and E). Confocal immunofluorescence showed that Snail-1 expression was mainly nuclear, and paralleled higher levels of phospho-MEK in Cav1^−/−^ MCs (Fig4F). CI-1040 reduced the expression of Snail-1 transcript and protein in WT and Cav1^−/−^ MCs, accompanied by a drop in the level of phospho-MEK (Fig4D–F and Supplementary Fig S2A). Nuclear localization of SMAD2/3 was higher in Cav1^−/−^ MCs than in WT (Fig4G). Interestingly, CI-1040 significantly reduced SMAD2-3 nuclear staining. To further explore the effect of MEK-ERK signaling on SMAD nuclear localization and activity, we transfected the untransformed mesothelial cell line MeT-5A with a luciferase reporter construct containing multiple SMAD3 binding sites or with a reporter containing the *id1* promoter, which specifically binds SMAD1-5. CI-1040 reduced TGF-β-stimulated SMAD3-driven luciferase expression and C-terminal phosphorylation (linked to transcriptional activity), whereas it had the opposite effect on basal and stimulated activity of SMAD1-5 (Supplementary Fig S2B–D). Interestingly, Cav1 knockdown increased expression of FN and PAI-1, controlled by SMAD3, and of Snail-1. In the reverse experiment, expression of these proteins was reduced upon overexpression of lentivirally encoded Cav1 (Supplementary Fig S2E).

These findings are in good agreement with the quantitative proteomics analysis of MEF-derived extracellular matrices, which showed an increase in abundance of proteins directly or indirectly related to TGF-β activity such as fibromodulin, gremlin-1, LTBP2 (latent-transforming growth factor beta-binding protein 2), SPARC, metalloproteases, serpins, and thrombospondin-1 in matrices from Cav1^−/−^ mice (Fig3). These results show that Cav1 deficiency in MCs increases Snail-1 expression and SMAD3 nuclear translocation, and suggest that MEK, in addition to controlling the ERK-Snail-1 pathway, also affects SMAD function, enhancing SMAD2-3 activity while reducing that of SMAD1-5.

### Acquisition of a spindlelike cell morphology and increased permeability by Cav1^−/−^ MCs depend on the MEK-ERK1/2 pathway

**Figure 5:**
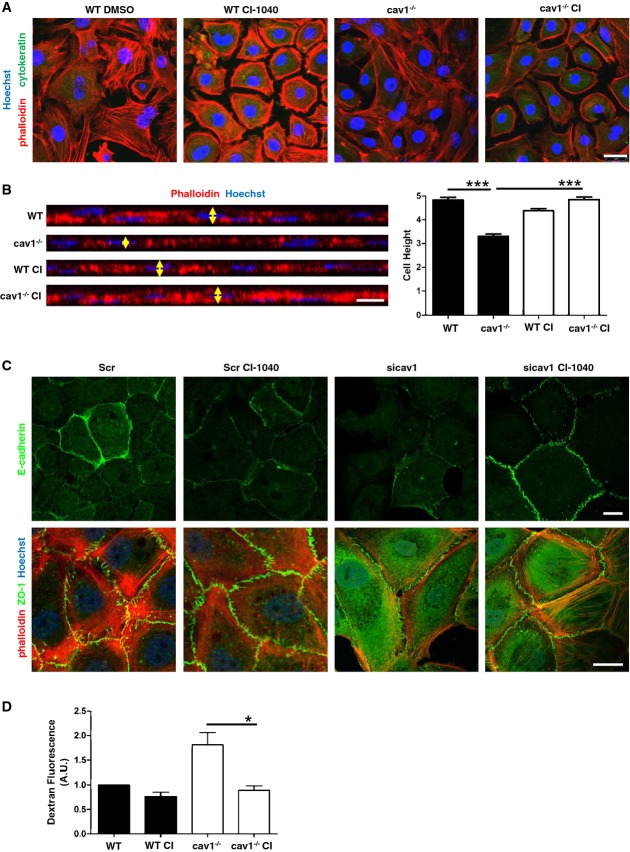
Morphological, biochemical, and functional features in the absence of Cav1 depend on ERK1/2 pathway Confocal IF of phalloidin and cytokeratin expression and localization in MCs from WT (left) and Cav1^−/−^ mice (right). Cells were treated with 2 μM CI-1040 or DMSO for 48 h. Fixed and permeabilized cells were stained with phalloidin (F-actin) and immunostained for cytokeratin. Cell nuclei were stained with Hoechst 33342. Scale bar: 25 μm. Images show the results of a representative independent experiment of three performed. For quantification of elliptical factor, see Supplementary Fig S3A.Left: Representative *X*–*Z* images are shown from the experiment described in (A). Arrows indicate the maximal apical dimensions used to calculate cell height. Right: quantification of cell height in the experiment described above. Scale bar: 10 μm. ***P <* *0.001.Confocal IF of E-cadherin (top), actin, and ZO-1 expression (bottom) in HPMCs transfected with control or Cav1-targeting siRNAs and then treated for 48 h with DMSO or with 2 μM CI-1040. Nuclei were stained in the bottom images with Hoechst 33342 (blue). Images show the results of a representative independent experiment of three performed. For quantification of intensity, see Supplementary Fig S3B. Scale bar: 15 μm.Permeability of WT and Cav1^−/−^ MC monolayers. Cells grown on filter inserts were pretreated with DMSO or CI-1040 (2 μM) for 1 h, and the permeability to fluorescent dextran was monitored over time (40-min values shown). The image shows the result of 8 experiments performed. Scale bar: 15 μm. *P =* *0.027.

MCs from Cav1^−/−^ mice have a spindlelike morphology compared with the typical cobblestone epithelial appearance of WT MC cultures (Fig5A, for a quantification see Supplementary Fig S3A). Moreover, MCs from Cav1^−/−^ mice show flattened morphology with reduced cell height, suggesting acquisition of a mesenchymal-like phenotype (Fig5B) (Mukhina *et al*, 2004). Treatment of Cav1^−/−^ MCs with CI-1040 induced reversion to an epithelial-like phenotype in MCs from mice. We reproduced this experiment in Cav1 silenced HPMCs. Cav1 knockdown induced disappearance of E-cadherin and ZO-1 from membrane junctions, whose levels were significantly restored upon treatment with CI-1040 (Fig5C, for a quantification see Supplementary Fig S3B).

The reduced expression and altered localization of E-cadherin and other proteins located at cell junctions in the absence of Cav1 imply functional alterations of cell permeability, an important function of MCs during peritoneal dialysis. This was confirmed by *ex vivo* analysis of dextran leakage, which revealed a markedly higher permeability in Cav1^−/−^ MC cultures that was reverted by treatment with CI-1040 (Fig5D).

### Enhanced migration and invasion by Cav1^−/−^ MCs is dependent on MEK-ERK1-2

**Figure 6:**
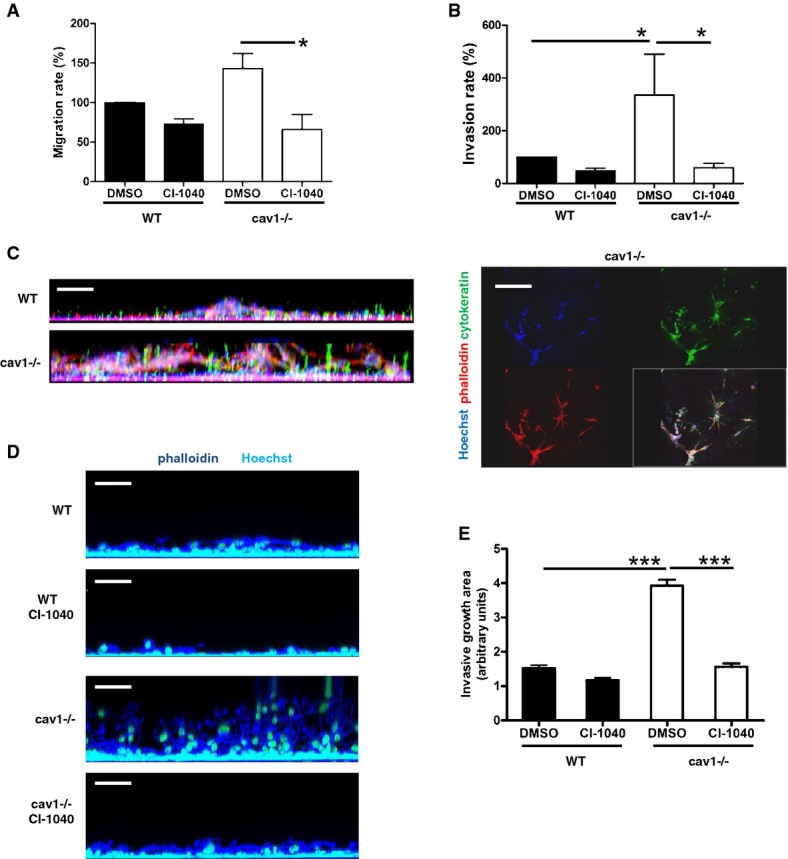
Migratory and invasive phenotype of Cav1^−/−^ MCs depends on ERK1/2 pathway Effect of MEK inhibition on MC migration through transwell filters. MCs from WT and Cav1^−/−^ mice were pretreated with DMSO or CI-1040 (2 μM) for 24 h and allowed to migrate for 24 h through transwell filters. Migrating cells were fixed and nuclei-counted in ten fields per sample using a fluorescence microscope (40×  magnification). Each experiment was performed in duplicate, and 5 experiments were performed. *P =* *0.03.Effect of MEK inhibition on MC invasion through type I collagen-coated transwell filters. Cells were treated as above. *P =* *0.016 for WT versus Cav1^−/−^ MCs and *P =* *0.010 for DMSO- versus CI-1040-treated Cav1^−/−^ MCs.Effect of MEK inhibition on three-dimensional invasion by WT and Cav1^−/−^ MCs. MCs were pretreated (24 h) with DMSO or CI-1040 (2 μM) and then overlaid with a matrigel+collagen matrix. Invasion was monitored over 24 h. Three-dimensional invasion was enhanced by adding 10% FCS to the well. Cells were fixed and stained with anti-cytokeratin (green), phalloidin (red), and Hoechst 33342 (cell nuclei; blue). Left: *xz* maximal projection; right: *xy* acquisition at the top of the well (Cav1^−/−^ MCs). Scale bar: 100 μm. Each experiment was performed in duplicate, and 5 experiments were performed.Full 3D invasion experiment. Hoechst-stained nuclei are shown in yellow; phalloidin in blue. Scale bar: 50 μm.Quantification of MC invasion of 3D matrix. Each experiment was carried out in triplicate, and three experiments were performed. ***P <* *0.001 for WT versus Cav1^−/−^ MCs and ***P <* *0.001 for DMSO- versus CI-1040-treated Cav^−/−^ MCs.

The acquisition of migratory and invasive capacities is typical of cells undergoing EMT (Thiery *et al*, 2009). To investigate the effect of Cav1 on these properties, we analyzed MC migration through transwell filters. MCs from Cav1^−/−^ mice migrated more efficiently than WT cells through uncoated filters (Fig6A), and a more pronounced effect was obtained in assays of invasiveness across filters coated with type I collagen (Fig6B), indicating that MCs lacking Cav1 acquire the capacity to degrade ECM, a characteristic of cells that have undergone EMT (Thiery *et al*, 2009). In both assays, the migratory/invasive advantage of Cav1^−/−^ MCs was abolished by CI-1040.

Cav1^−/−^ MCs also showed a greater invasive capacity than WT cells in a 3-dimensional invasion assay on matrigel matrices (Fig6C). In this assay, bundles of cytokeratin-positive (green) Cav1^−/−^ cells were detected emerging from the top of the gel, ruling out the possibility that the invasiveness of Cav1^−/−^ cells was due to contamination of the MC preparation with fibroblasts. As with the other assays, invasion through matrigel by Cav1^−/−^ MCs was blocked by CI-1040 (Fig6D and E).

### Endothelium of Cav1-deficient mice has a mesenchyme-like phenotype

**Figure 7:**
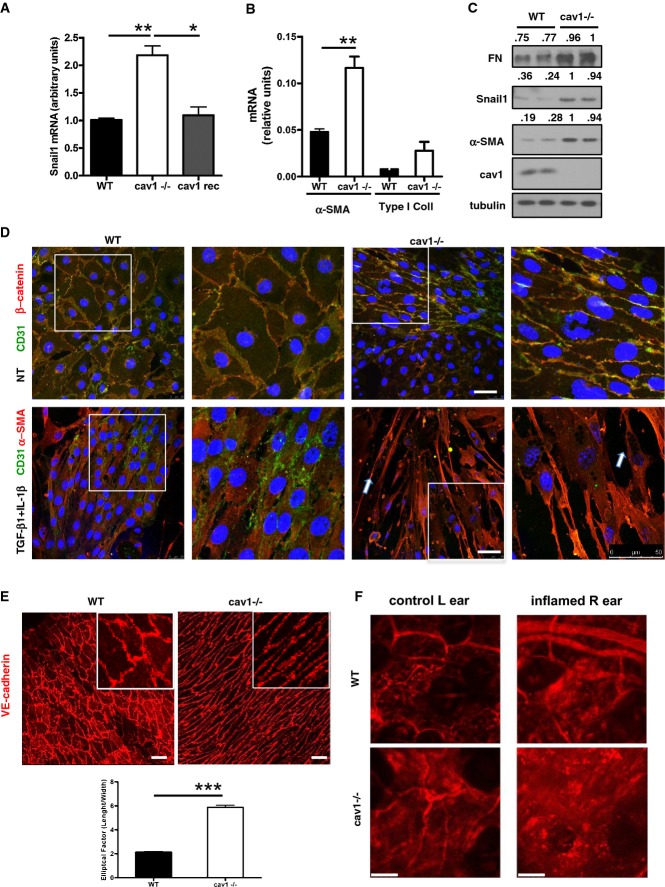
EMT-like status of mouse lung endothelial cells (MLEC) from Cav1^−/−^ mice The levels of Snail-1 transcript in WT, Cav1^−/−^ or Cav1 reconstituted (Cav1 rec) immortalized MLECs. mRNA was amplified from total RNA by quantitative RT–PCR; histone H3 mRNA expression was used for normalization. Bars represent means ± SEM of duplicate determinations in four independent experiments. **P =* *0.003; *P <* *0.033.The levels of α-SMA and type I collagen transcripts in WT or Cav1^−/−^ MLECs. PCR amplification and normalization as in (A); **P =* *0.002. Bars represent means ± SEM of duplicate determinations in four independent experiments.Western blot for FN, α-SMA, Snail-1, and Cav1 in total lysates of primary MLECs by WB from WT and Cav1^−/−^ mice. Tubulin was detected as a loading control. Data are representative of three independent experiments.Top: Confocal IF of primary MLECs from WT (left) and Cav1^−/−^ mice (right). Cells were fixed and stained for CD31 (green) and β-catenin (red). Bottom: Confocal IF of primary MLECs from WT (left) and Cav1^−/−^ mice (right). Cells were stimulated with TGF-β1 (2 ng/ml) in combination with IL-1β (0.5 ng/ml) for 48 h. Cells were fixed and stained for CD31 (green) and α-SMA (red). Nuclei were stained with Hoechst 33342 (blue). Representative images are shown of one experiment of three performed. Scale bar: 50 μm. The adherence to ICAM2 coated beads, used for MLEC purification, (see arrows), confirms the endothelial origin of these cells.Confocal IF of aorta sections from WT and Cav1^−/−^ mice. Aortas were fixed *in vivo*, sectioned and stained with monoclonal anti-VE-cadherin. Scale bar: 30 μm. Bottom: quantification of elliptical factor with MetaMorph. ***P <* *0.001. Representative images are shown of one experiment of five performed.Intravital imaging of paravascular permeability in ears of WT (top) and of Cav1^−/−^ mice (bottom) under steady state (left) or inflammatory conditions (right). TRITC-dextran was used as vascular tracer. The images shown are representative of five independent experiments. Scale bar: 50 μm.

Mouse lung endothelial cells (MLEC) share a mesodermal origin with MCs and have similar morphological and functional properties; moreover, both cell types express abundant Cav1, which is reduced by treatment with TGF-β1 (Igarashi *et al*, 2009). Like MCs, MLECs undergo a transition to a mesenchymal phenotype (EndMT) upon exposure to pro-fibrotic and inflammatory stimuli or shear stress (Zeisberg *et al*, 2007; Egorova *et al*, 2011; Medici & Kalluri, 2012). EndMT induction has been directly linked to Snail-1 expression (Kokudo *et al*, 2008), and we found that immortalized Cav1^−/−^ MLECs upregulated Snail-1 expression, an effect reversed upon Cav1 reconstitution (Fig7A). Consistent with induction of EndMT, primary Cav1^−/−^ MLECs expressed higher levels of α-SMA, type I collagen, and FN than cells from WT mice (Fig7B and C).

In addition, and resembling the results obtained with MCs, MLECs from Cav1^−/−^ mice had a spindlelike morphology in *ex vivo* culture (Fig7D) and acquired a fibroblastoid shape when treated with TGF-β1 in combination with IL-1β, a costimulation known to induce full EMT in HPMCs (Yanez-Mo *et al*, 2003; Strippoli *et al*, 2008). Moreover, a polarized endothelial-cell morphology was also evident in whole-mount staining of Cav1^−/−^ aorta (Fig7E). These morphological changes were accompanied by increased endothelial permeability (estimated from vessel dye leakage), both in steady state and in response to aptene-induced inflammation (Fig7F).

### CI-1040 prevents fibrosis, EMT, and altered peritoneal membrane function in Cav1^−/−^ mice undergoing PD

**Figure 8:**
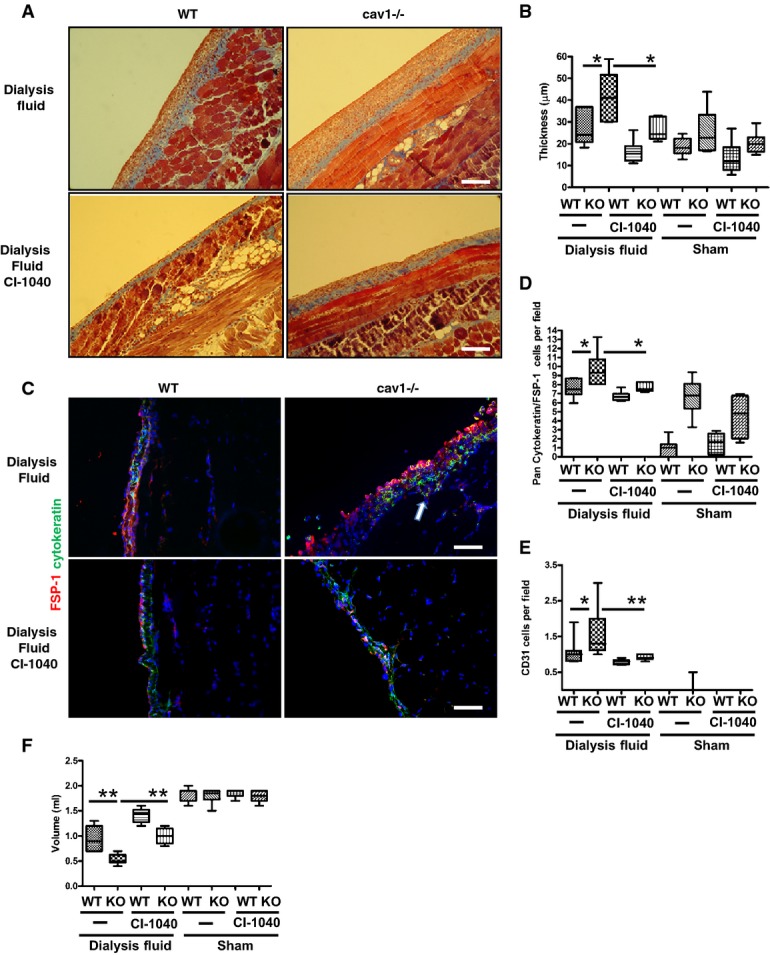
The MEK inhibitor CI-1040 reverses thickening, fibrosis, and angiogenesis in the parietal peritoneum of Cav1^−/−^ mice upon exposure to PD fluid WT or Cav1^−/−^ mice were exposed to peritoneal dialysis (PD) fluid or sham 5 times per week for three weeks with contemporaneous treatment with CI-1040 (200 mg/kg) or vehicle. Eight mice were analyzed per group. Representative hematoxylin and eosin staining of parietal peritoneum sections. Scale bar: 50 μm.Quantification of peritoneal membrane thickness. *P =* *0.035 for WT versus Cav1^−/−^ exposed to peritoneal dialysis fluid; *P =* *0.04 for Cav1^−/−^ exposed to pertoneal dialysis fluid versus Cav1^−/−^ exposed to peritoneal dialysis fluid and treated with CI-1040.Representative IF of parietal peritoneal sections stained for pan-cytokeratin (green) and FSP-1 (red), and with DAPI to visualize cell nuclei. Scale bar: 30 μm. *P *=* *0.03; *P* = 0.01. Arrow shows cytokeratin-FSP-1 double-positive MCs invading the submesothelial stroma.Quantification of parietal peritoneal EMT as the number of double-positive pancytokeratin- and FSP-1-stained cells.Quantification of CD31-positive cells; *P =* *0.038; **P =* *0.005.Results of a 30-min peritoneum equilibration test performed on the last day of the experiment (see Materials and Methods) to measure total peritoneal volumes. **P =* *0.0023 for WT versus Cav1^−/−^ exposed to peritoneal dialysis fluid; **P =* *0.0043 for Cav1^−/−^ exposed to peritoneal dialysis fluid versus Cav1^−/−^ exposed to peritoneal dialysis fluid and treated with CI-1040.

To study the role of Cav1 in the peritoneal EMT and fibrosis that occurs in patients undergoing PD, we used a mouse model of PD (Aroeira *et al*, 2008), in which mice are instilled daily with glucose-based PD fluids over a three-week period. Masson's trichrome staining of the parietal peritoneum of control animals revealed a thicker submesothelial space in Cav1^−/−^ mice, and this thickening was significantly increased by exposure to PD fluids (Fig8A and B). As expected, ERK1/2 activation was higher in peritoneal tissue from Cav1^−/−^ mice exposed to PD fluids with respect to WT tissue (Supplementary Fig S4A). Similar to TGF-β1 stimulation (Fig1A), exposure to PD fluid markedly reduced Cav1 levels in *ex vivo* cultured MCs from WT mice (Supplementary Fig S4B).

The induction of EMT in response to PD fluids was monitored by immunofluorescence analysis of parietal peritoneal sections for cytokeratin (MC marker) and FSP-1 (used as a marker of myofibroblasts and EMT conversion). Exposure of WT and Cav1^−/−^ mice to PD fluids caused discontinuity and loss of the cytokeratin-positive MC monolayer (Fig8C, top). However, sections from Cav1^−/−^ mice contained higher numbers of cytokeratin-FSP-1 double-positive MCs invading the submesothelial stroma (Fig8C and D) and of CD31-positive cells in the sub-mesothelial space, indicating increased angiogenesis in Cav1^−/−^ mice (Fig8E, see arrow).

Treatment with CI-1040 during the period of PD fluid exposure abolished ERK1/2 phosphorylation in WT and Cav1^−/−^ mice (Supplementary Fig S4C), and markedly reduced peritoneal thickening and EMT (Fig8A–D). Moreover, CI-1040 blocked the increase in CD31-positive cells in the peritoneum of PD-fluid-treated Cav1^−/−^ mice (Fig8E). The cell-division Ki67 marker was not significantly increased in Cav1^−/−^ peritoneum upon exposure to PD fluid, indicating that the increased peritoneal fibrosis and angiogenesis in these animals were not directly related to increased cell proliferation during the analysis period (Supplementary Fig S5).

The functional impact of the observed morphological changes in the peritoneum was assessed by a peritoneal equilibration test on the last day of treatment. Mice from all groups were injected with 1.5 ml PD solution, and total peritoneal volumes were recovered 30 min later. The volumes recovered from PD-fluid-exposed Cav1^−/−^ mice were significantly lower than those recovered from WT mice, indicating increased permeability in the peritoneum of Cav1^−/−^ mice (Fig8F). The permeability alterations in Cav1^−/−^ mice were significantly inhibited by co-treatment with CI-1040.

These results demonstrate a major role of MEK/ERK1/2 signaling in the events leading to EMT and fibrosis during PD, and identify Cav1 as an important regulator of these events.

### Ectopic expression of Cav1 in MCs from PD patients reverses EMT

**Figure 9:**
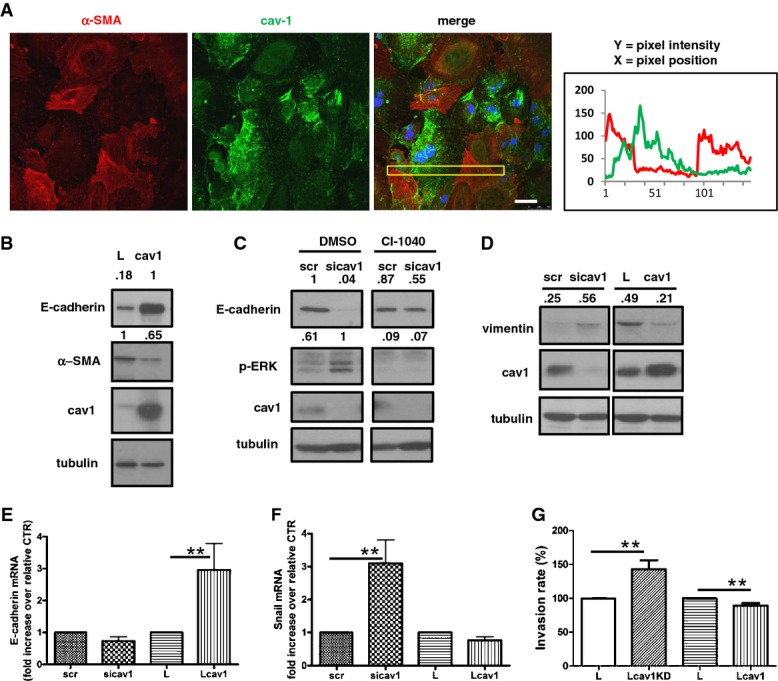
Cav1 ectopic expression reverses EMT in human peritoneal MCs from PD patients Confocal IF of α-SMA (red) and Cav1 (green) in HPMCs from the peritoneal effluent of patients undergoing PD. Representative image from eight independent experiments from eight different patients. Scale bar: 50 μm.Western blot for E-cadherin, α-SMA, and Cav1 in whole-cell lysates of effluent-derived HPMCs infected with control (L) or Cav1-expressing lentivirus (Cav1). Tubulin was detected as a loading control. Data are representative of three independent experiments.Western blots showing the expression of E-cadherin, phospho-ERK, and Cav1 in whole-cell lysates of effluent-derived HPMCs transfected with control (scr) or Cav1-targeting siRNAs and treated with 2 μM CI-1040 or DMSO. Tubulin was detected as a loading control. Data are representative of three independent experiments.Western blot showing expression of vimentin and Cav1 in effluent-derived HPMCs transfected with Cav1 siRNAs (left) or infected with Cav1 lentivirus (right). Data are representative of three independent experiments.E-cadherin mRNA expression in effluent-derived HPMCs expressing Cav1 siRNAs or lentivirally encoded Cav1. Quantitative RT–PCR was performed on total RNA. Histone H3 mRNA was used for normalization. Bars represent means ± SEM of duplicate determinations in six independent experiments from six patients. **P =* *0.002.Snail-1 mRNA expression in effluent-derived HPMCs expressing Cav1 siRNAs or lentivirally encoded Cav1. PCR and normalization as in (E). Bars represent means ± SEM of duplicate determinations in five independent experiments from five patients. **P =* *0.008.Effect of Cav1 silencing or ectopic expression on invasion by effluent-derived HPMCs. MCs expressing lentivirally encoded Cav1 shRNA (LCav1KD) or Cav1 protein (LCav1) were allowed to invade for 24 h through type I collagen-coated transwell filters. Invading cells were fixed and nuclei were counted in ten fields per sample using a fluorescence microscope (40× magnification). Each experiment was performed in duplicate, and six experiments were performed from six patients. **P =* *0.002. Source data are available online for this figure.

HPMCs isolated from the effluent of patients undergoing PD are a useful model for the analysis of the changes occurring in the peritoneal HPMC monolayer *in vivo* in patients undergoing EMT (Del Peso *et al*, 2008). Immunostaining of these cells for Cav1 revealed a heterogeneous pattern, with Cav1 expression inversely correlating with the expression of α-SMA (Fig9A). To evaluate the role of Cav1 in the EMT status of these cells, we overexpressed lentivirally encoded Cav1, which resulted in rescue of E-cadherin expression and a decrease in α-SMA levels (Fig9B). Consistent with these findings, E-cadherin expression in patient-derived HPMCs was further reduced by Cav1 knockdown, and this effect was partially rescued by treatment with CI-1040 (Fig9C). Furthermore, Cav1 knockdown increased vimentin levels, which were again reduced upon ectopic Cav1 expression (Fig9D). The direct correlation between Cav1 and E-cadherin expression and the inverse correlation between Cav1 and Snail-1 expression were confirmed by RT–PCR under the same experimental settings (Fig9E and F). Cav1 knockdown also significantly increased the invasive capacity of patient-derived HPMCs on collagen-coated transwell filters, whereas ectopic Cav1 expression in the same cells had the opposite effect (Fig9G). Interestingly, exposure of HPMCs to dexamethasone or tamoxifen, two drugs endowed with anti-inflammatory or anti-fibrotic activity, respectively, led to increased Cav1 levels, and a concomitant increase in E-cadherin levels and a reduction in those of α-SMA and vimentin (Supplementary Fig S6A–C)(Jang *et al*, 2013; Loureiro *et al*, 2013).

## Discussion

While Cav1 has been widely studied for its role in endocytosis and cell signaling organization, its possible implication in EMT-related fibrosis has been scarcely addressed (Gvaramia *et al*, 2013). The results presented here establish a pivotal role for Cav1 in the EMT associated with PD-induced PM deterioration and demonstrate the effectiveness of inhibiting the MEK-ERK1/2 pathway in blocking or reversing these events. Lack of Cav1 in human and mouse MCs is linked to acquisition of a pre-EMT phenotype characterized by loss of the ‘cobblestone’ epithelial morphology, a switch from epithelial to mesenchymal marker expression, ECM production, and an enhanced migratory and invasive capacity. Proteomics analysis of MEF-derived extracellular matrices revealed increased abundance of ECM proteins and of proteins related to enhanced TGF-β1 activity in matrices produced by MEFs from Cav1^−/−^ mice. These changes correlate with peritoneal thickening and permeability in Cav1^−/−^ mice and with activation of ERK1/2, Snail-1 expression, and SMAD2-3 nuclear localization, and all pre-EMT phenotypic features are blocked or reversed by the MEK inhibitor CI-1040.

Similar changes occur in Cav1^−/−^ endothelial cells. Moreover, EMT, angiogenesis, and peritoneal permeability induced by PD fluid are more severe in Cav1^−/−^ mice and are blocked by CI-1040. The clinical relevance of these findings is confirmed by the ability of ectopic Cav1 to reverse EMT in MCs from PD patients, whereas EMT is exacerbated by Cav1 suppression.

The reduced expression or/and altered localization of intercellular junction proteins in the absence of Cav1 are consistent with defective function of the peritoneal membrane as a semi-permeable barrier during PD. This is supported by the *ex vivo* assays showing high permeability of Cav1^−/−^ MC monolayers compared with WT cells. This may be related to the EMT-like state of Cav1^−/−^ MCs, characterized by a loosening of cell junctions. Accordingly, *in vivo* permeability assay detected microvascular leakage in Cav1^−/−^ mice, confirming previous studies showing that lack of Cav1 causes microvascular hyperpermeability due to hyperactivation of endothelial nitric oxide synthase (eNOS) (Schubert *et al*, 2002; Murata *et al*, 2007). Other studies reported enhanced transvascular protein transport in Cav1^−/−^ mice, probably reflecting passive filtration across large pores in a context of increased microvascular pressure (Rosengren *et al*, 2006; Grande *et al*, 2009). Our *ex vivo* results suggest that MC monolayer permeability is altered per se, in the absence of elevated microvascular pressure. The increased permeability observed in the microvasculature of Cav1^−/−^ mice may also contribute to the altered functional properties of the PM during PD, a hypothesis reinforced by the peritoneal equilibration test performed on the last day of the treatment of mice with PD fluids; the lower volumes recovered from the peritoneum of Cav1^−/−^ mice suggest a higher peritoneal permeability in these mice.

Mesothelial and endothelial cells have a common mesodermal origin and share morphological and functional features. Moreover, like MC EMT, TGF-β-induced EndMT requires Snail-1 (Kokudo *et al*, 2008). Our experiments show that Cav1 suppresses α-SMA and Snail-1 expression in primary and immortalized MLECs, and Cav1^−/−^ MLECs and *in situ* aortic endothelium are spindle-shaped, suggesting EndMT onset. Interestingly, Cav1 has been demonstrated to regulate the expression of junction-associated proteins in brain microvascular endothelial cells (Song *et al*, 2007). Our study is further supported by the recent observation that Cav1 deficiency leads to increased α-SMA and Snail-1 expression in mouse pulmonary ECs *in vitro* (Li *et al*, 2012). Overall, our results suggest that Cav1 controls biochemical, morphological and functional parameters linked to EndMT.

There are several possible mechanisms through which Cav1 might regulate the establishment of EMT and fibrosis in the peritoneal membrane. Cav1, through its scaffolding domain (CSD), interacts with and downregulates serine and tyrosine kinases such as Src, AKT, and H-Ras, thus controlling cell cycle and regulating gene expression (Couet *et al*, 1997; Murata *et al*, 2007). The lack of direct evidence for interaction between the CSD and caveolin-binding motifs (CBM) in Cav1-interacting proteins has challenged the CSD/CBM hypothesis (Collins *et al*, 2012); however, understanding of the effect of Cav1 or CSD on signaling pathways is far from complete (Parton & Del Pozo, 2013). For some proteins, such as those belonging to the Ras family of GTPases and to the Src family of kinases, Cav1 might regulate membrane versus cytoplasmic localization, and thus activity, by controlling associations with liquid-ordered PM domains (del Pozo *et al*, 2005; Norambuena & Schwartz, 2011). A candidate mechanism for Cav1-mediated downregulation of MEK1 activity is cooperation with the endogenous Ras/MAPK inhibitor Dok1 to promote MEK1 localization in the cytosol (Burgermeister *et al*, 2011). The recent observation that Cav1 may modulate MEK-ERK1/2 signaling pathway through organization of Ras microclusters (Ariotti *et al*, 2014) fits with our results, emphasizing the role of Cav1 in the regulation MEK-ERK1/2-driven EMT.

Previous studies of the role of Cav1 in EMT were mostly conducted in experimental models of cancer. Lu *et al* (2003) demonstrated in an *in vitro* system that chronic EGF treatment of carcinoma cell lines resulted in transcriptional Cav1 downregulation and induction of Snail-1, correlating with downregulation of E-cadherin expression and increased invasion of collagen gels. The inverse relationship between Cav1 and Snail-1 was recently confirmed in a model of pancreatic cancer, and the same study also showed the correlation between Cav1 and E-cadherin expression (Salem *et al*, 2011). In an ovarian carcinoma setting, ectopic expression of Cav1 stabilized adherens junctions through inhibition of Src-related kinases (Miotti *et al*, 2005). Snail-1, in addition to directly targeting E-cadherin expression, downregulates other proteins associated with cell junctions, such as claudins and occludin, with knock-on effects on the expression of other proteins such as metalloproteinases, integrins and ECM proteins (Thiery *et al*, 2009). Snail-1 can alter cell polarity through repression of Crumbs and can control cell scattering and invasiveness (Haraguchi *et al*, 2008; Whiteman *et al*, 2008; Stanisavljevic *et al*, 2011). In a non-tumoral setting, Snail-1 overexpression is sufficient to induce renal fibrosis (Boutet *et al*, 2006).

**Figure 10:**
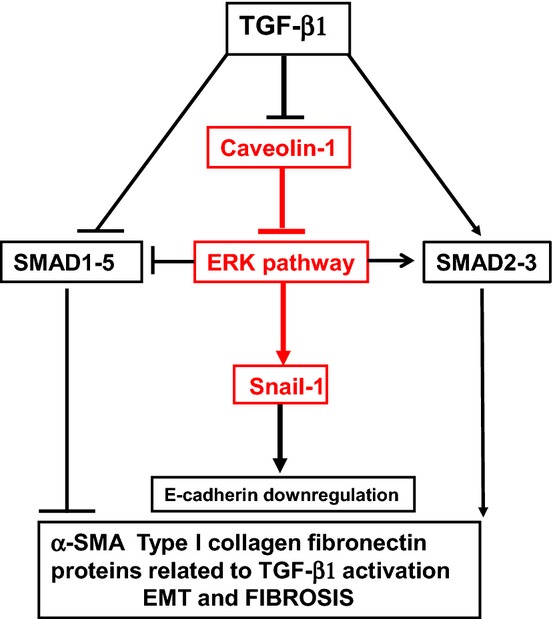
Control of EMT-related signalling pathways by Cav1 Cav1 regulates peritoneal EMT and fibrosis through an action on ERK and SMAD pathways.

Our study emphasizes the central role of MEK-ERK1/2 suppression in Cav1-mediated regulation of EMT and fibrosis. In agreement with our results, ERK1/2 hyperactivation has been previously demonstrated in Cav1^−/−^ mice and is reduced by Cav1 re-expression (Engelman *et al*, 1998; Wang *et al*, 2006). Activated ERK1/2 in the absence of Cav1 might trigger EMT by targeting the transcription factor Egr1 to the Snail-1 promoter, thus enhancing Snail-1 transcriptional activity (Grotegut *et al*, 2006). However, Cav1 may also regulate EMT independently of MEK/ERK1/2 signals. Cav1 dampens SMAD2-3 signaling through an interaction with TGF-β1 type 1 receptors (Razani *et al*, 2001b), and TGF-β1 type 1 and type 2 receptors are internalized in Cav1-containing vesicles that target them to the degradation pathway (Di Guglielmo *et al*, 2003). Cav1 modulates SMAD2 and SMAD3 nuclear accumulation in fibroblasts in an experimental model of idiopathic pulmonary fibrosis (Wang *et al*, 2006). Another possibility is that, in addition to a direct effect on SMADs, Cav1 deficiency indirectly modifies TGF-β1-driven SMAD activation through a MAPK-mediated effect. In our study, MEK inhibition dampened SMAD3 nuclear translocation while enhancing SMAD1-5-8 signaling. Previous studies demonstrated that ERK phosphorylation of the SMAD1 polylinker region inhibits SMAD1 nuclear accumulation and transcriptional activity (Kretzschmar *et al*, 1997). More recently, ERK activation was shown to increase the half-life of C-terminal phosphorylation of SMAD2 and SMAD3 and to increase their transcriptional activity (Hough *et al*, 2012). This complex interrelationship of Cav1 with the MEK/ERK and SMAD pathways in the regulation of mesothelial cell EMT is summarized in Fig10. De-repression of MEK-ERK-Snail-1 signaling pathway by Cav1 deficiency directly promotes E-cadherin downregulation, a hallmark of EMT. ERK1/2 hyperactivation further unbalances the SMAD2-3/SMAD1-5 pathways, favoring the pro-fibrotic SMAD2-3 pathway.

The structure of the visceral and parietal peritoneum is similar in humans, rats, and mice (Nishino *et al*, 2007), suggesting that experiments in genetically modified mice can provide valuable information about the damage associated with clinical PD. Here, we present what is to our knowledge the first analysis of the effect of PD-fluid exposure in a genetically modified animal model. Our results suggest that lack of Cav1 causes a series of changes leading to increased invasion by trans-differentiated MCs of the sub-mesothelial stroma, fibrosis, and altered peritoneal permeability. The abnormal migratory and invasive capacities of Cav1^−/−^ MCs were furthermore confirmed in *ex vivo* migration and invasion assays.

The detection of an EMT-like phenotype in Cav1-deficient mesothelial and endothelial cells indicates that both cell types are likely to participate in the establishment of peritoneal tissue damage and functional alteration during PD. TGF-β1 and VEGF produced by MCs during PD might induce EC proliferation and EndMT (Lai *et al*, 2000; Aroeira *et al*, 2005), and the subsequently increased solute diffusion through leaky EC junctions might contribute to the progression of tissue fibrosis in the sub-mesothelial stroma.

An important finding of our study is the ability of the MEK inhibitor CI-1040 to block EMT and fibrosis in mice exposed to PD fluids. MEK inhibition is an active focus of oncology research, but less attention has been paid to its role in acute and chronic inflammation, despite the important role of MEK/ERK1/2 signals in the induction and resolution of inflammation (Chappell *et al*, 2011). Our findings are consistent with others showing that CI-1040 reduces kidney fibrosis and TGF-β1 expression in a mouse model of chronic allograft nephropathy (Wang *et al*, 2008). In another study, CI-1040 reduced IL-1β production and ECM alterations in a mouse model of arthritis (Thiel *et al*, 2007). CI-1040 has been replaced for clinical studies by new MEK inhibitors, due mainly to concerns about physical properties and bio-availability; however, no concerns have been raised about this drug's specificity, and therefore, information obtained with it provide a foundation for future work with new and more potent MEK inhibitors (Barrett *et al*, 2008; Chappell *et al*, 2011).

The clinical relevance of our results is further demonstrated by the experiments HPMCs from the effluent of PD patients. These cells, having undergone EMT *in vivo*, present low levels of E-cadherin, high levels of α-SMA, and ERK1/2 activation (Yanez-Mo *et al*, 2003; Strippoli *et al*, 2008). Ectopic expression of Cav1 in these cells increased E-cadherin and reduced α-SMA levels. Rescue of E-cadherin in post-EMT human cells *in vivo* could have important impacts on structure and signaling because E-cadherin limits pathways that lead to EMT induction, such as NF-κB and β-catenin pathways, and contributes to Snail-1 inhibition (Solanas *et al*, 2008). This conclusion is supported by our finding that silencing Cav1 in post-PD HPMCs further increased their invasiveness, whereas this was significantly reduced upon ectopic Cav1 expression. Interestingly, the fact that Cav1 levels may be increased by drugs with anti-inflammatory and anti-fibrotic activity, such as dexamethasone or tamoxifen, amplifies the translational implications of these findings.

Our study establishes Cav1 as a major checkpoint in the transition of MCs and ECs from an epithelial toward a mesenchymal identity. The control of Cav1 levels in chronic inflammatory fibrotic pathologies is thus relevant to the understanding of chronic inflammation and also suggests possible therapeutic routes. The molecular mechanisms underlying EMT and fibrosis in the peritoneum may also be relevant to clinical issues unrelated to PD, such as the prevention of post-operative peritoneal adhesions and the control of peritoneal metastasis.

## Materials and Methods

### Mice

Cav1-deficient mice (STOCK Cav1tm1Mls/J; Razani *et al*, 2001a) and WT B6129SF2/J controls were from The Jackson Laboratory (USA) and were used for *in vivo* and *ex vivo* experiments. Cav1-deficient mice and WT littermates (B6.Cg-CAV1tm1mls/J; Drab *et al*, 2001) were used to confirm *ex vivo* experiments. Mice from both genders were equally used in the different experimental conditions. All animal procedures conformed to EU Directive 2010/63EU and Recommendation 2007/526/EC regarding the protection of animals used for experimental and other scientific purposes, enforced in Spanish law under Real Decreto 1201/2005, and were approved by CNIC animal care and ethics committee.

### Cells

Mouse peritoneal MCs were obtained by digestion of parietal peritoneum samples from WT and Cav1^−/−^ mice. The samples were digested with a 0.125% trypsin solution containing 0.01% EDTA for 15 min with occasional agitation at 37°C. Cells were cultured in DMEM F12 supplemented with 20% fetal calf serum, 50 U/ml penicillin, 50 μg/ml streptomycin, and 1% Biogro-2 (containing insulin, transferrin, ethanolamine, and putrescine) (Biological Industries, Beit Haemek, Israel). Biogro-2 is routinely added to favor *in vitro* MC differentiation, being washed out the day before performing the experiment.

To induce EMT, mouse MCs were treated with TGF-β1 (2 ng/ml) (R&D Systems, Minneapolis, MN) for the indicated times. HPMCs were obtained by digestion of omentum samples from patients who were undergoing unrelated abdominal surgery (Stylianou *et al*, 1990). The samples were digested with a 0.125% trypsin solution containing 0.01% EDTA for 15 min with occasional agitation at 37°C. Cells were cultured in Earle's 199 medium supplemented with 10% fetal calf serum, 50 U/ml penicillin, 50 μg/ml streptomycin, and 1% Biogro-2. To induce EMT, HPMCs were treated with TGF-β1 (1 ng/ml) as described previously (Yanez-Mo *et al*, 2003) (Aroeira *et al*, 2005). The cytokine dose used is in the range of those detected in peritoneal dialysis fluids from patients with peritonitis (Lai *et al*, 2000) and is similar to those used in previous studies (Yang *et al*, 1999; Yanez-Mo *et al*, 2003). The human mesothelial cell line MeT-5A (ATCC, Rockville, MD) was cultured in Earle's M199 as above.

Effluent-derived MCs were isolated from clinically stable PD patients as described previously (Lopez-Cabrera *et al*, 2006). To control for fibroblast contamination, the purity of HPMC and effluent-derived MC cultures was determined from the expression of the standard mesothelial markers intercellular adhesion molecule (ICAM)-1 and cytokeratin (Aroeira *et al*, 2007), (Strippoli *et al*, 2008, 2012). MCs from PD effluents generally express cytokeratins, although at low levels. MC cultures are generally negative for the endothelial marker CD31 and the pan-leukocyte marker CD45. Omental and peritoneal effluent-derived MCs were generally > 95% pure by FACS, and samples with > 5% contaminating cells were discarded. ICAM-1 expression was also used to monitor the purity of mouse MCs (Supplementary Fig S7). Effluent-derived MCs were cultured in Earle's M199 as above.

The study was approved by the ethics committee of the Hospital Universitario de la Princesa (Madrid, Spain). Written informed consent was obtained from all PD patients and from omentum donors prior to elective surgery.

MLECs were obtained and cultured as described (Oblander *et al*, 2005). Briefly, lungs from WT or Cav1^−/−^ mice were excised, disaggregated and digested in 0.1% collagenase (GIBCO) for 1 h at 37°C. The cell suspensions were seeded onto plates coated with 10 μg/ml fibronectin (Sigma), 10 μg/ml collagen I (PureCol) and 0.1% gelatin (Sigma). After attachment, cells were negatively selected with anti-CD16/CD32 mAb (BD Biosciences) coupled to magnetic beads (Dynal, Invitrogen) and then positively selected with magnetic-bead-coupled anti-ICAM-2 (BD Biosciences). Mouse embryonic fibroblasts (MEFs) were obtained from day 13.5 embryos and cultured essentially as described (Razani *et al*, 2001a).

### Antibodies and chemicals

Monoclonal antibodies against Snail-1, ERK1/2, phospho-ERK1/2, phospho-SMAD1-5-8, and Cav1 were from Cell Signaling Technology; monoclonal antibodies against E-cadherin, β-catenin, and γ-catenin were from BD (Becton-Dickinson Laboratories, Mountain View, CA); monoclonal antibodies against occludin was from Invitrogen (Carlsbad, CA); monoclonal antibodies against tubulin, α-SMA, α-catenin, vimentin, pan-cytokeratin, and fibronectin were from Sigma (Saint Louis, MO); monoclonal anti-N-cadherin and polyclonal anti-ZO-1 were from Zymed (Invitrogen, Carlsbad, CA); polyclonal anti-actin, -SMAD2-3, and -phospho-MEK were from Santa Cruz Biotechnology (CA); and polyclonal anti-phospho SMAD2-3 was from Biosource (Camarillo, CA). Monoclonal anti-CD54 was from Biolegend (San Diego, CA); polyclonal anti-Fsp1 was from Dako (Glostrup, Denmark); monoclonal anti-CD31 from Serotec (Oxford, UK); and polyclonal anti-Ki67 from Abcam (Cambridge, UK). Monoclonal anti-JAM-A clone BV-11 was a gift from Dr. E. Dejana (Milan Italy). Fluor 647?phalloidin and Hoechst 33342 were from Invitrogen (Carlsbad, CA). CI 1040 was from Selleck (Houston, TX).

### Western blotting

Cells were lysed, and Western blotting was performed as previously described (Strippoli *et al*, 2008).

### Confocal microscopy and immunofluorescence

Cells were fixed for 20 min in 3% formaldehyde in PBS, permeabilized in 0.2% Triton X-100/PBS for 5 min, and blocked with 2% BSA for 20 minutes. For E-cadherin staining, cells were fixed and permeabilized in ice-cold methanol for 5 min. Secondary antibodies (conjugated to Alexa-647, -488, and -541) were from Pierce Chemical Company (Rockford, IL, USA). Confocal images were acquired using a Leica SP5 spectral confocal microscope. The spectral technology allows discrimination between yellow and green fluorescence. In some experiments, Leica SP8 confocal microscope with stimulated emission depletion (STED) microscopy (super-resolution microscopy) was used.

### Flow cytometry

Cells were trypsinized, washed, and resuspended in PBS. Cells (1 × 10^5^) were incubated with 100 μl monoclonal anti-ICAM-1, washed with PBS and then incubated with 100 μl of a 1:50 dilution of FITC-conjugated anti-mouse Ig. Alternatively, 1 × 10^5^ cells were incubated with 100 μl of a 1:50 dilution of FITC-conjugated monoclonal anti-CD45 for 20 min at 4°C and washed with PBS. Fluorescence was measured using a FACScan® flow cytometer (Becton Dickinson Labware, Lincoln Park, NJ, USA).

### siRNA-mediated Cav1 knockdown and lentiviral infection

MCs (1.2 × 10^5^) were seeded on 24-well plates 24 h prior transfection. Cells were transfected overnight with 80 pmol of siRNA corresponding to human Cav1 bases 403–423 (target sequence: AAGAGCTTCCTGATTGAGATT) or the same amount of control siRNA in 400 μl antibiotic-free medium containing 1 μl Dharmafect 1 (Dharmacon, Lafayette, CO) (Beardsley *et al*, 2005). Transfections were repeated after 48 h. 72 h after the last transfection, knockdown efficiency was determined by Western blot and cells were processed as indicated. An alternative Cav1 siRNA was designed (5′-GACGTGGTCAAGATTGACTTT-3′) corresponding to human Cav1 bases 254–277. This sequence was cloned into pLVX-shRNA2, which contains a ZsGreen1 reporter (Clontech, Mountain View, CA). Lentiviral infection was performed as in Goetz *et al* (2011). MCs were also infected with pRR-CMV-Cav-IRES-GFP with pLVX-Cav-ZsGreen, or with empty vectors as a control.

### Cell transfection and luciferase assays

Smad3 transcriptional activity was measured by transient transfection of MeT-5A cells with the PAI-1 reporter plasmid (van Zonneveld *et al*, 1988) and SMAD1–5–8 activity by transfection with BRE-luc reporter plasmid (Korchynskyi & ten Dijke, 2002). Cells (2 × 10^5^) were transfected with 2 μg of reporter plasmid together with 500 ng of pRL-null, which contains a promoterless Renilla luciferase gene (Promega, Madison, WI). For transfections, cells were incubated for 4 h with a 1:2.5 w/v (ng/μl) mix of DNA and Lipofectamine (Lipofectamine 2000; Invitrogen, Carlsbad, CA, USA) in serum-free medium. Cells were subsequently pretreated overnight with vehicle (DMSO) or CI-1040 (2 μM) and stimulated with TGF-β1 for 9 h. Luciferase activity was measured by the dual-luciferase reporter assay system (Promega) using a Sirius single tube luminometer (Berthold Detection Systems GmbH, Pforzheim, Germany). All experiments were performed in triplicate.

### Reverse-transcriptase polymerase chain reaction

Total RNA was extracted with the RNeasy kit (Qiagen GmbH, Hilden, Germany), and cDNA was obtained from 500 ng RNA with the Omniscript RT kit (Qiagen). Quantitative PCR was carried out in a LightCycler (Roche Diagnostics GmbH, Mannheim, Germany) using a SYBR Green kit (Roche Diagnostics GmbH) and the following specific primer sets: 5′TGAAGGTGACAGAGCCTCTG3′ and 5′TGGGTGAATTCGGGCTTGTT3′ for E-cadherin; 5′GCAAATACTGCAACAAGG3′ and 5′GCACTGGTACTTCTTGACA3′ for Snail-1; 5′GCTATGATGAGAAATCAACCG3′ and 5′GCTTCCCCATCATCTCCATTC3′ for type I collagen; 5′CCTGAAGCTGAAGAGACTTGC3′ and 5′CGTTTCTCCGACCACATAGGA3′ for fibronectin; 5′GCGACCCTAAACACCTCAAC3′ and 5′ATGCCGTCAAAACTGTGTGTC3′ for Cav1; 5′ F aggaaatggctcgtcaccttcgtgaata3′ and 5′ ggagtgtcggttgttaagaactagagct3′ for vimentin; 5′AAAGCCGCTCGCAAGAGTGCG3′ and 5′-ACTTGCCTCCTGCAAAGCAC3′ for histone H3 (used for normalization); 5′CAAGTATCAGGGTCAAGTGCC3′ and 5′CCAAATCCGATACGTGATCTTC3′ for mouse E-cadherin; 5′AGCTGGCCAGGCTCTCGGTG3′ and 5′GCAGCCAGACTCTTGGTGCTT3′ for mouse Snail-1; 5′GCAAACCTATAGCTGAGAAGTG3′ and 5′CAAGTACAGTCCACCATCATC3′ for mouse FN; 5′CAGTCGCYGTCAGGAACC3′ and 5′GTGCTGTCTTCCTCTTCACAC3′ for mouse α-SMA; 5′TGCCGCGACCTCAAGATGTG3′ and 5′CACAAGGGTGCTGTAGGTGA3′ for mouse type I collagen; and 5′AAAGCCGCTCGCAAGAGTGCG3′ and 5′-ACTTGCCTCCTGCAAAGCAC3′ for histone H3. All experiments were carried out in duplicate. After amplification, PCR products were confirmed by melting-curve analysis and gel electrophoresis.

### Preparation of cell-derived extracellular matrices and quantitative proteomics analysis

Cell-derived extracellular matrices were prepared as previously described (Castello-Cros & Cukierman, 2009; Goetz *et al*, 2011). The resulting soluble protein extracts from ECM preparations were digested following the whole-proteome in-gel digestion method (Bonzon-Kulichenko *et al*, 2011) using modified porcine trypsin (Promega) at a final ratio of 1:20 (trypsin–protein). Digestion proceeded overnight at 37°C in 100 mM ammonium bicarbonate, pH 8.8. A total of 200 μg of each peptide mixture were labeled with different iTRAQ tags according to the manufacturer's protocol, desalted, and joined together.

The resulting tryptic peptide mixtures were subjected to nano-liquid chromatography coupled to mass spectrometry analysis for protein identification and quantification on a Q-Exactive mass spectrometer (Thermo Fisher, San José, CA, USA). An enhanced FT-resolution spectrum (resolution = 70.000) followed by the MS/MS spectra from most intense fifteen parent ions were analyzed along the chromatographic run (272 min). See Field *et al* (2013) for details. Protein identification from tandem mass spectra was done using Sequest™ running under Proteome Discoverer 1.4.0.288 (Thermo Fisher Scientific). Sequest results were analyzed using the probability ratio method (Martinez-Bartolome *et al*, 2008) and filtered out using the refined FDR method (Jorge *et al*, 2009). Protein quantification from reporter ion intensities and statistical analysis of quantitative data were performed using QuiXoT, as described (Jorge *et al*, 2009; Navarro *et al*, 2014).

### *In vitro* permeability assay

The permeability of mouse MC monolayers was measured with the Millipore *In Vitro* Permeability Assay kit (Chemicon). Cells (100,000) were seeded on the membranes of the chambers provided. After 48 h, dextran-FITC was added to the upper chamber and was recovered after 40 min from the bottom chamber and measured in a fluorimeter.

### Invasion assays

For 3-dimensional invasion assays, cells (2 × 10^5^) were treated with CI-1040 (2 μM) or vehicle for 12 h and then seeded in Ibidi 15-well chambers and allowed to attach for 3 h. 40% matrigel (10 μl) in combination with type I collagen (30 μg/ml) in serum-free medium was laid over the cells. After 1 h, 50 μl full medium containing 20% serum was added and cells incubated for 20 h. Cells were fixed with 3% paraformaldehyde (PFA), permeabilized with 0.25% Triton PBS, and stained for 12 h with rhodamine-phalloidin, anti-cytokeratin and Hoechst in PBS. Confocal images were captured with a Leica SP5 fitted with a 40× oil objective. Maximum projection images consist of 65 individual images with a z-distance of 120 μm (*n* = 8, *P-value < 1 × 10E-5). Alternatively, migration and invasion assays were performed using polycarbonate inserts with a 8-μm pore size (Costar, Cambridge, MA). For invasion assays, inserts were precoated with 40 μl type I collagen (300 μg/ml) (PureCol, Inamed, Fremont, Canada) and incubated overnight at 37°C to allow gel formation. MCs were pretreated for 24 h with DMSO or CI-1040 (2 μM) in 10% FBS M-199 medium. MCs (5 × 10^4^) in 100 μl assay medium (M-199 0% FBS) were added to the upper chamber. Invasion stimulus (10% FBS) was added to the lower chamber in 600 μl assay medium. After 24 h, inserts were fixed with 4% PFA, non-invading cells on the upper face of the membrane were removed with a cotton swab, filters were cut, and nuclei of invading cells were stained with Hoechst 33342. Invading cells were counted in ten fields per sample using a fluorescence microscope (40× magnification). Each experiment was carried out in duplicate, and at least 5 experiments were performed.

### Whole-mount staining of mesenterium

Live mice anesthesized with a mixture of zoletil-dontor were surgically opened and perfused with 1% PFA in PBS at 7 ml/min, using a programmable syringe (Harvard Apparatus). Ventral mesenterium was collected and post-fixed in 1% PFA for 1 h at room temperature (RT). Tissues were stained as described in Baluk *et al* (2007). Briefly, tissues were incubated overnight (O/N) at RT with PBS containing 0.3% Triton X-100, 5% goat serum (Jackson ImmunoResearch) and primary antibody. Samples were then thoroughly washed with 0.3% Triton X-100 in PBS and stained with appropriate secondary antibodies O/N at RT. Secondary antibody was Alexa488 donkey anti-rat. After final washes, labeled samples were post-fixed in 1% PFA for 30 min and mounted for microscopy in Prolong® Gold antifade reagent (Molecular Probes). Confocal z-stacks up to 100 μm in depth were obtained using a LSM 700 laser-scanning microscope equipped with a LD LCI Plan/Apochromat 25x/0.8 Imm Korr DIC M27 objective (Zeiss). Images were processed to obtain maximal projections, fluorescence intensity profiles, and colocalization data (Imaris, Volocity and ImageJ softwares).

### Intravital imaging of paravascular permeability

Ears were taped to the center of a coverslip and attached with high vacuum grease. Dibutylphtalate (1:1 dissolved in acetone) was applied topically in both sides of the ear 24 h prior to imaging to induce a cutaneous inflammatory response. Hairless areas were examined using a HCX PL APO lambda blue 20.0 × 0.70 IMM UV objective (multi-immersion, glycerol) coupled to an inverted microscope (DMI6000; Leica) equipped with a confocal laser-scanning unit (TCS-SP5; Leica). Non-invasive intravital imaging procedures were performed in a thermostatic chamber at 37°C. For short-term studies (1–2 h), animals were first anesthesized with i.p. ketamine (50 mg/kg), xylazine (10 mg/kg), and acepromazine (1.7 mg/kg), and repeated half-doses were administered when needed over the course of the experiments. Vessels were traced with 100 μl 70 kDa TRITC-dextran (Molecular Probes) dissolved in PBS at concentration 70 μM. Optimal confocal sections (spaced 0.63 μm along the z-axis) were obtained and processed using Imaris version 7.3.1 (Bitplane). Mean fluorescence intensity analysis was performed using LAS AF Software (Leica Microsystems).

### PD fluid exposure in mice

Sixty-four 12- to 16-week-old mice were used (32 WT B6129SF2/J and 32 Cav1^−/−^ Cav1tm1Mls/J). PD fluid or saline solution was instilled via a peritoneal catheter connected to an implanted subcutaneous mini access port under isoflurane (MTC Pharmaceuticals) anesthesia (Access Technologies, Skokie, IL, USA) (Loureiro *et al*, 2011). Animals were implanted with the peritoneal access port to receive 0.2 ml of saline with 1 IU/ml of heparin during the first week after surgery, to facilitate wound healing. Thereafter, four groups of 16 mice (each of them formed by 8 WT and 8 Cav1^−/−^ mice, matching gender, weight, and age) were instilled during a 4-week period, 5 days per week as follows: group A received 1.5 ml standard PD fluid composed of lactate-buffered 4.25% glucose (StaySafe, Fresenius, Bad Homburg, Germany); group B received 1.5 ml standard PD fluid contemporaneously with oral CI-1040 (200 mg/kg per day); group C received 1.5 ml saline solution; and group D received saline solution contemporaneously with 200 mg/kg CI-1040. CI-1040 was administered orally with a syringe and was dissolved in full-fat milk to promote absorption (Lorusso *et al*, 2005). Food and water were provided *ad libitum* during the experiment, and animals were housed in individually ventilated cages, 4 animals/cage. On the final day of treatment, all animals underwent a peritoneal equilibrium test. Mice were instilled with 2 ml PD solution and were anesthetized 30 min later with isoflurane (MTC Pharmaceuticals, Cambridge, Ontario, Canada) and sacrificed. Total peritoneal volumes were recovered, and parietal peritoneum samples were obtained from the side contralateral to the implanted catheter.

### Statistical analysis

Statistical significance was determined with a *t*-test or the nonparametric Mann–Whitney rank-sum *U*-test with OriginPro7 software (OriginLab Co.) or GraphPad Prism version 4.0 (La Jolla, CA, USA). Differences were considered significant at *P* < 0.05.

**The paper explained**

**Problem**

Peritoneal dialysis (PD) is an alternative to hemodialysis for the treatment of end-stage renal disease. During PD, the peritoneal membrane (PM) acts as a permeable barrier across which ultrafiltration and diffusion take place. The number of patients included in PD programs has progressively increased worldwide, and is now used by 10–15% of the global dialysis population. Several studies have confirmed equivalent effectiveness, mortality and fluid balance status compared with hemodialysis, at least for the first 4–5 years. In addition, PD offers major advantages in terms of quality of life, cost, and opportunities for home-based treatment. However, chronic exposure to hyperosmotic, hyperglycemic and acidic dialysis solutions, as well as episodes of peritonitis and hemoperitoneum, can cause acute and chronic inflammation and injury to the PM, which undergoes progressive EMT (epithelial to mesenchymal transition of the PM mesothelium) and subsequent fibrosis, as well as angiogenesis and vasculopathy, eventually leading to discontinuation of PD. There is thus a high clinical priority in understanding the underlying molecular mechanisms that cause EMT—the first step towards fibrosis—as well as the identification of new potential tools to counteract the onset of peritoneal fibrosis is of high clinical relevance.

**Results**

This study analyzes the role of caveolin-1 (Cav1), a key structural component of endocytic and mechanoresponsive caveolae, in the induction of EMT and subsequent peritoneal fibrosis, and the molecular mechanisms involved. The peritoneum of Cav1^−/−^ mice showed increased EMT, thickness and fibrosis, which was further increased upon exposure to PD fluids. Lack of Cav1 was associated with hyperactivation of a MEK-ERK1/2-Snail-1 pathway that regulates the Smad2-3/Smad1-5-8 balance. Pharmacological blockade of MEK rescued E-cadherin and ZO-1 localization at inter-cellular junctions, reduced fibrosis, and restored peritoneal function in Cav1^−/−^ mice exposed to PD fluids. Moreover, PD-patient-derived mesothelial cells that had already undergone EMT *in vivo* reacquired epithelial features upon treatment with drugs increasing Cav1 levels or ectopic expression of Cav1.

**Impact**

Our study establishes Cav1 as a major checkpoint in the transition from an epithelial toward a mesenchymal identity in the peritoneum. The efficacy of MEK pharmacological inhibitors in counteracting the EMT/fibrosis developed in Cav1^−/−^ mice during PD warrants further translational studies. Moreover, our studies establish MEK inhibition as a candidate rationale for therapy of other pathological conditions not related to PD, such as post-operative adhesions, and other non-peritoneal fibrotic inflammatory diseases.

## Supporting Information

Source Data for Supplementary Figure S6

Review Process File
