# Supplementary material for: Caveolin-1 deficiency induces a MEK-ERK1/2-Snail-1-dependent epithelial–mesenchymal transition and fibrosis during peritoneal dialysis
Source: EMBO Mol Med. 2014 Dec 30;7(1):102–23. doi: 10.15252/emmm.201404127 (PMC4309670; doi:10.15252/emmm.201404127)
Supplement: Supplementary file 1 [file emmm0007-0102-sd1.pptx]

## Slide 1
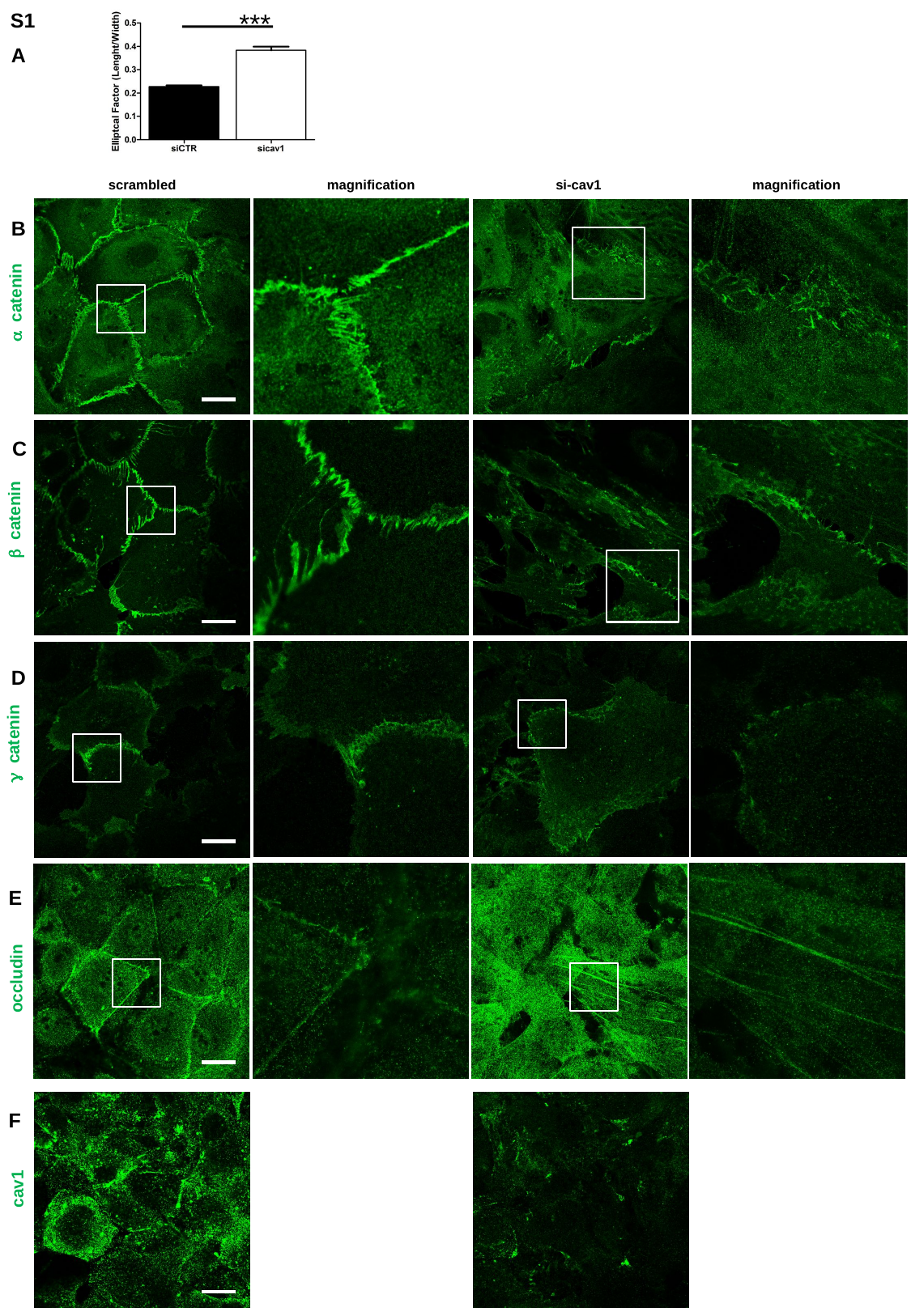

***
S1
A
scrambled
magnification
si-cav1
magnification
B
a catenin
C
b catenin
D
 g catenin
E
occludin
F
cav1
