# Supplementary material for: Caveolin-1 deficiency induces a MEK-ERK1/2-Snail-1-dependent epithelial–mesenchymal transition and fibrosis during peritoneal dialysis
Source: EMBO Mol Med. 2014 Dec 30;7(1):102–23. doi: 10.15252/emmm.201404127 (PMC4309670; doi:10.15252/emmm.201404127)
Supplement: Supplementary file 2 [file emmm0007-0102-sd2.pptx]

## Slide 1
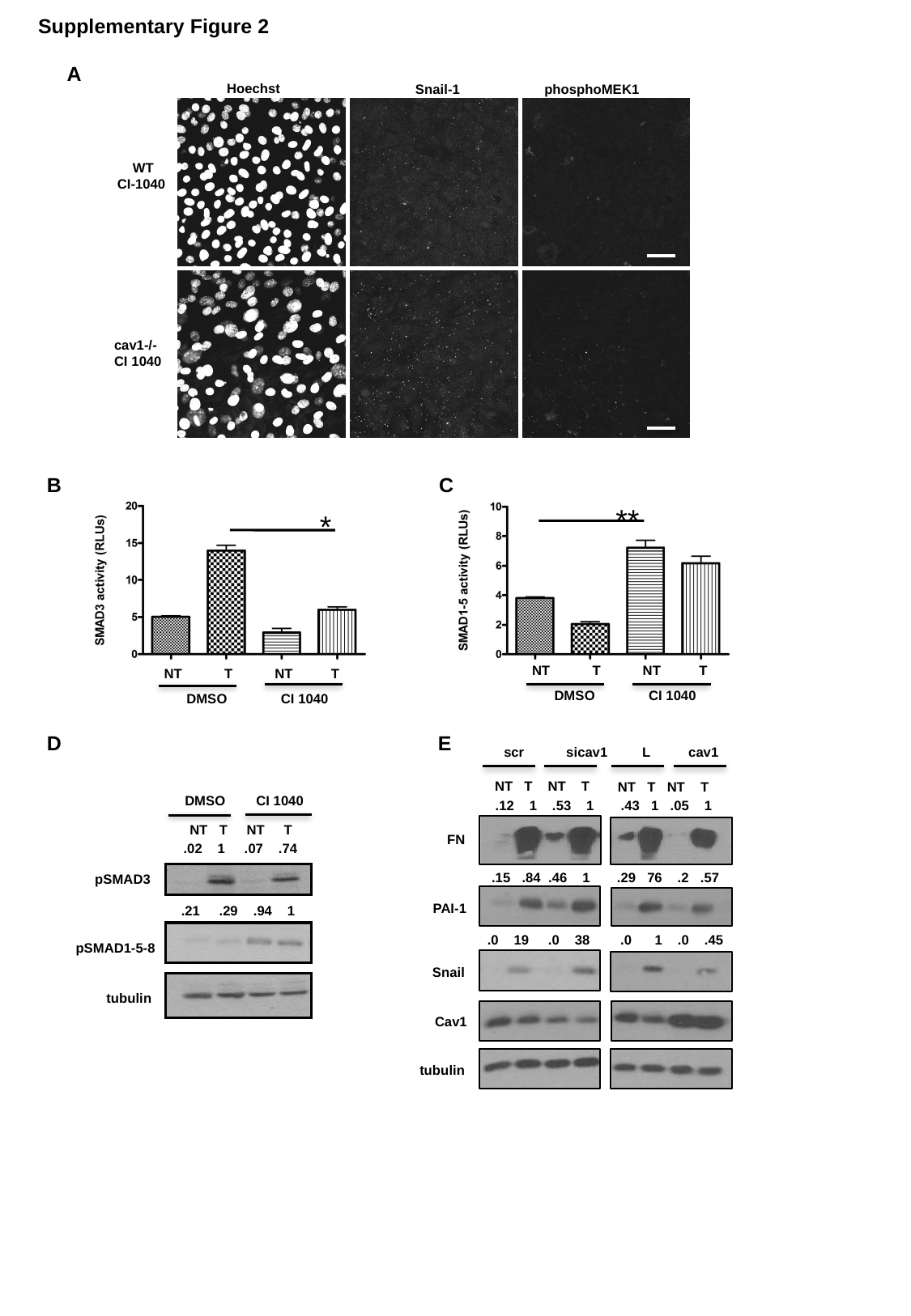

Supplementary Figure 2
A
Hoechst
phosphoMEK1
Snail-1
 WT
CI-1040
A
cav1-/-
CI 1040
B
C
*
NT T NT T
DMSO CI 1040
**
NT T NT T
DMSO CI 1040
D
E
 scr sicav1 L cav1
NT T NT T
NT T NT T
DMSO CI 1040
.12 1 .53 1 .43 1 .05 1
NT T NT T
FN
.02 1 .07 .74
.15 .84 .46 1 .29 76 .2 .57
pSMAD3
PAI-1
.21 .29 .94 1
.0 19 .0 38 .0 1 .0 .45
pSMAD1-5-8
Snail
tubulin
Cav1
tubulin
