# Supplementary material for: Caveolin-1 deficiency induces a MEK-ERK1/2-Snail-1-dependent epithelial–mesenchymal transition and fibrosis during peritoneal dialysis
Source: EMBO Mol Med. 2014 Dec 30;7(1):102–23. doi: 10.15252/emmm.201404127 (PMC4309670; doi:10.15252/emmm.201404127)
Supplement: Supplementary file 3 [file emmm0007-0102-sd3.pptx]

## Slide 1
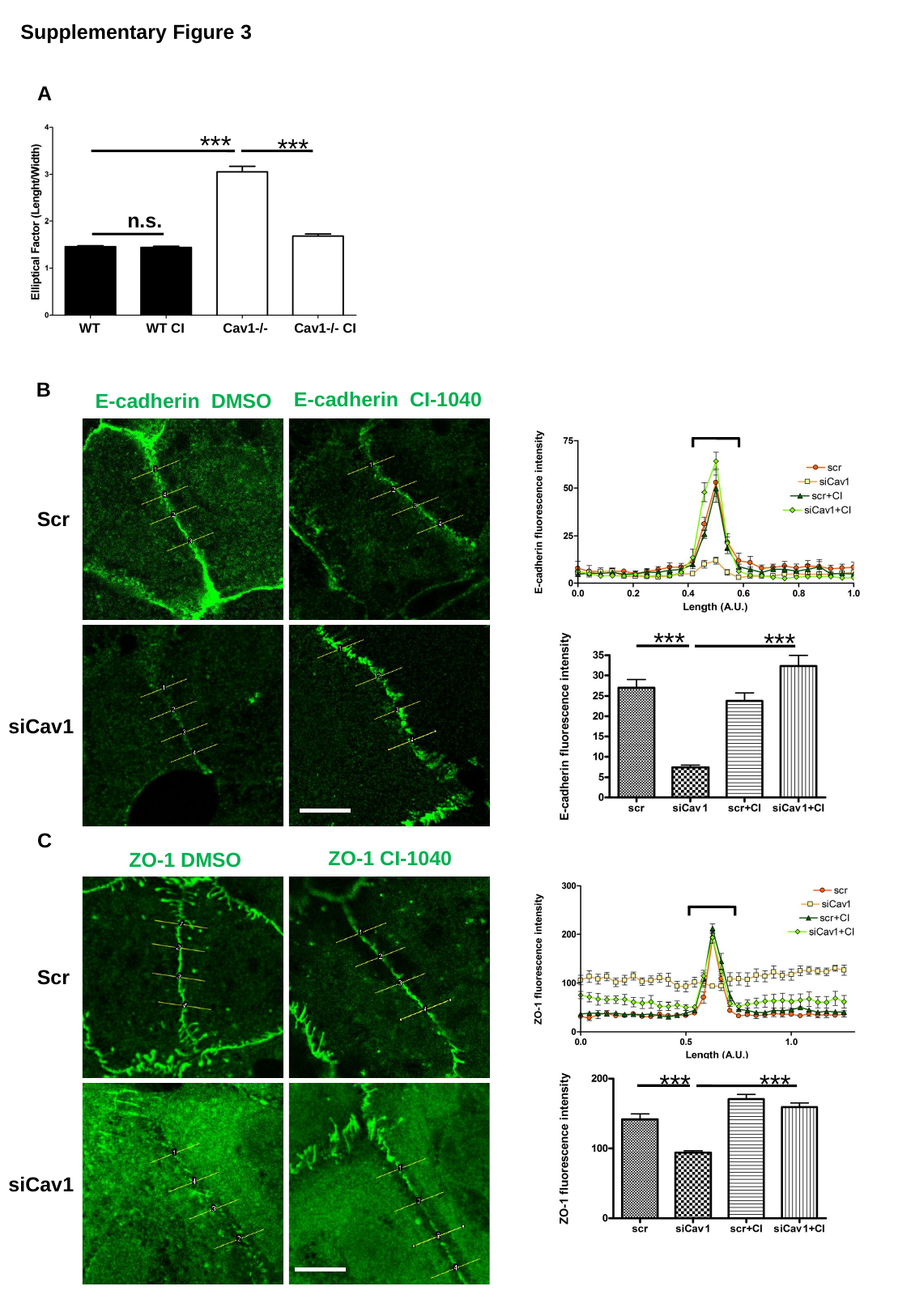

Supplementary Figure 3
A
***
 WT
 WT CI
 Cav1-/-
 Cav1-/- CI
***
n.s.
B
E-cadherin CI-1040
E-cadherin DMSO
Scr
***
***
siCav1
C
ZO-1 CI-1040
ZO-1 DMSO
Scr
***
***
siCav1
