# Supplementary material for: Caveolin-1 deficiency induces a MEK-ERK1/2-Snail-1-dependent epithelial–mesenchymal transition and fibrosis during peritoneal dialysis
Source: EMBO Mol Med. 2014 Dec 30;7(1):102–23. doi: 10.15252/emmm.201404127 (PMC4309670; doi:10.15252/emmm.201404127)
Supplement: Supplementary file 4 [file emmm0007-0102-sd4.pptx]

## Slide 1
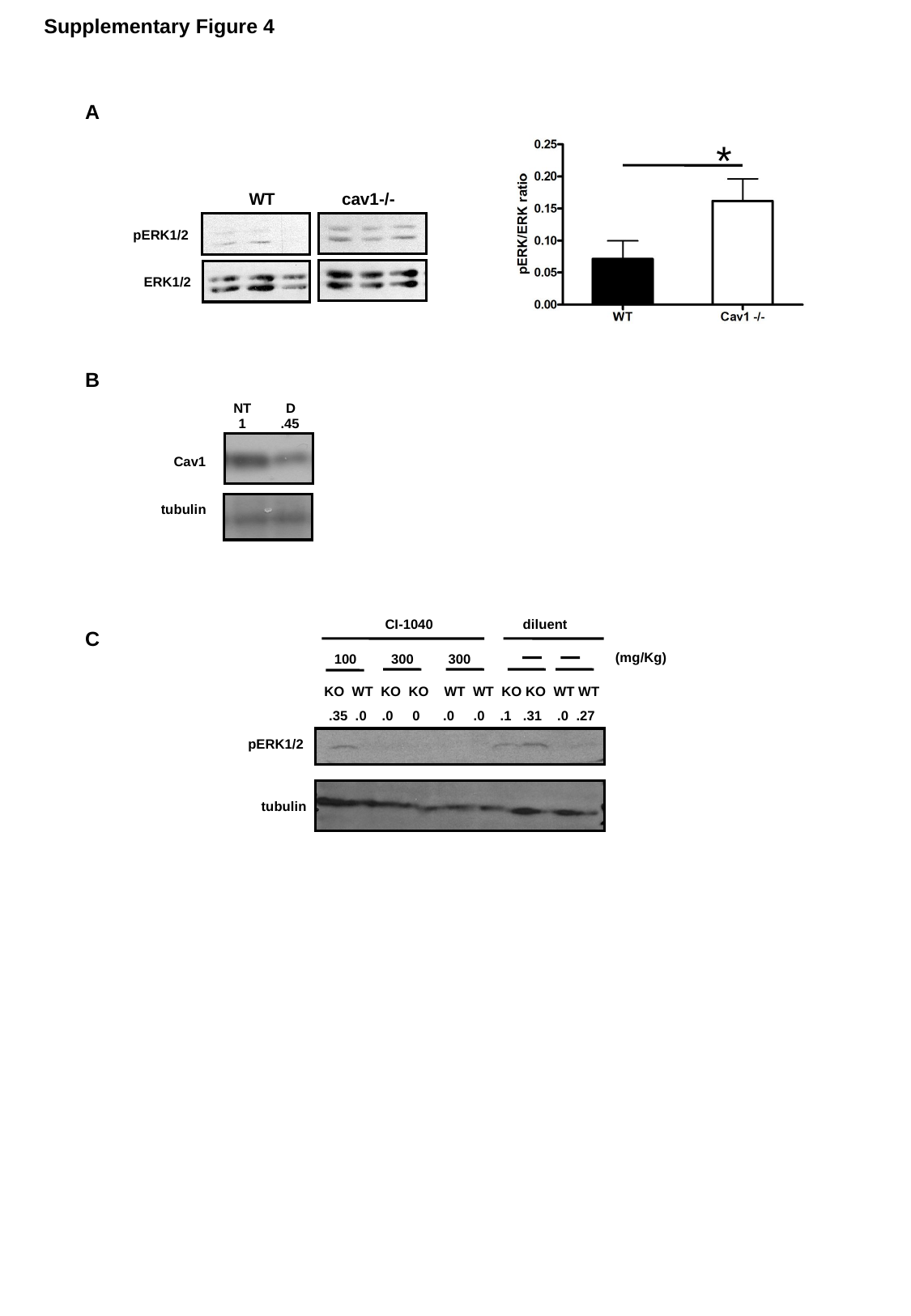

Supplementary Figure 4
A
*
 WT
cav1-/-
pERK1/2
ERK1/2
B
 NT D
 1 .45
Cav1
tubulin
CI-1040
diluent
C
(mg/Kg)
100 300 300
KO WT KO KO WT WT KO KO WT WT
 .35 .0 .0 0 .0 .0 .1 .31 .0 .27
pERK1/2
tubulin
