# Supplementary figures and images for: Caveolin-1 deficiency induces a MEK-ERK1/2-Snail-1-dependent epithelial–mesenchymal transition and fibrosis during peritoneal dialysis
Source: EMBO Mol Med. 2014 Dec 30;7(1):102–23. doi: 10.15252/emmm.201404127 (PMC4309670; doi:10.15252/emmm.201404127)

## Slide 1
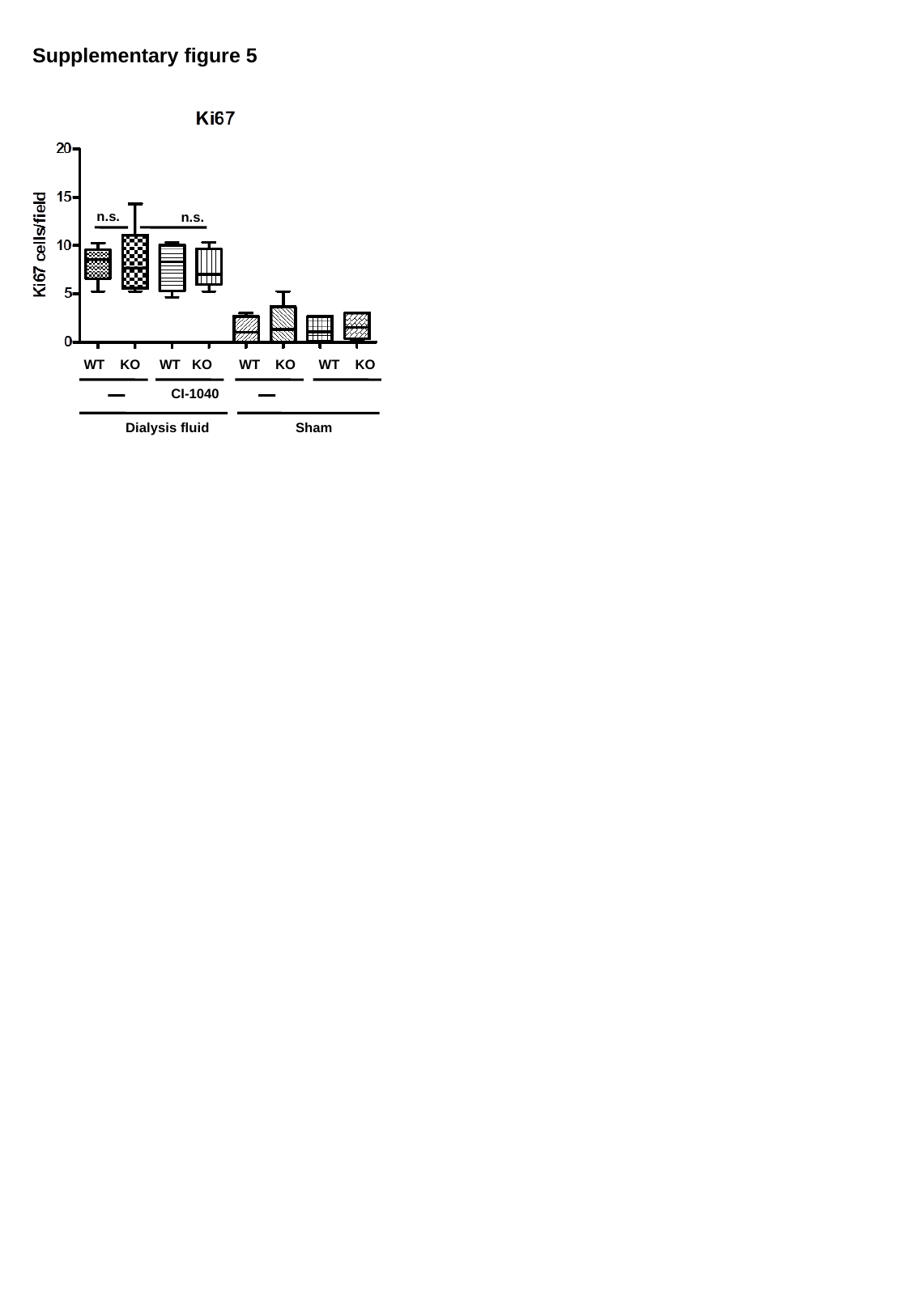

Supplementary figure 5
WT KO WT KO WT KO WT KO
CI-1040
Dialysis fluid
Sham
n.s.
n.s.

Supplement: Supplementary file 5 [file emmm0007-0102-sd5.pptx]

Supplementary Figure 2

D

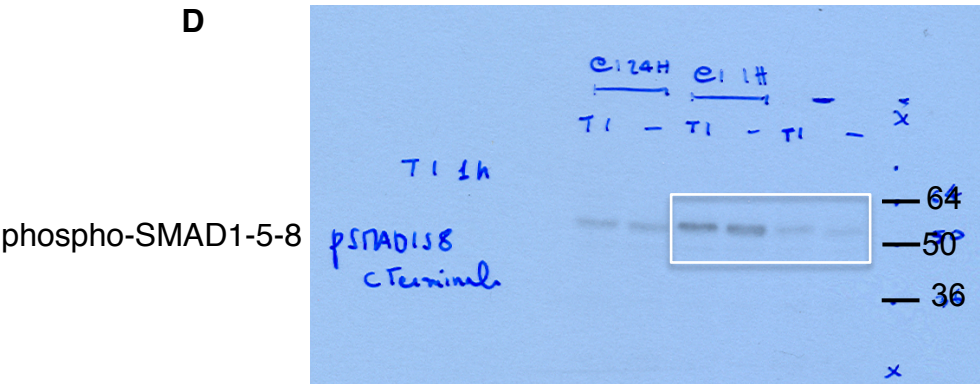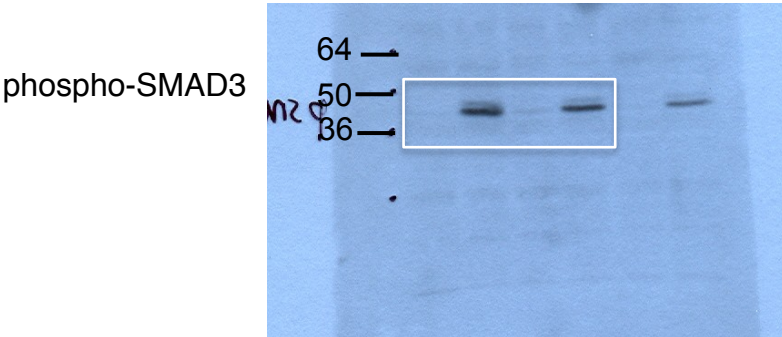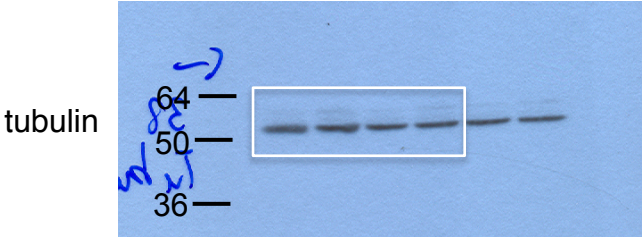

E

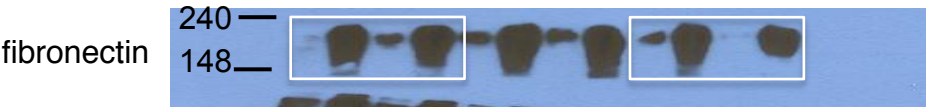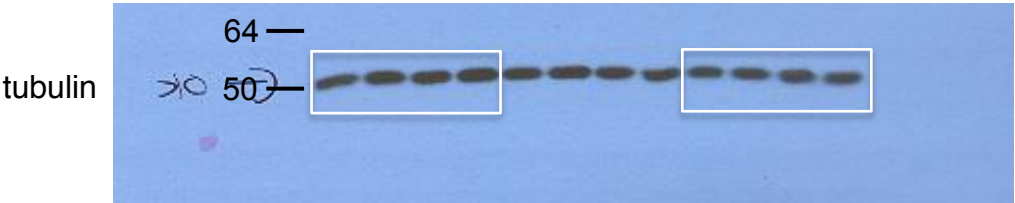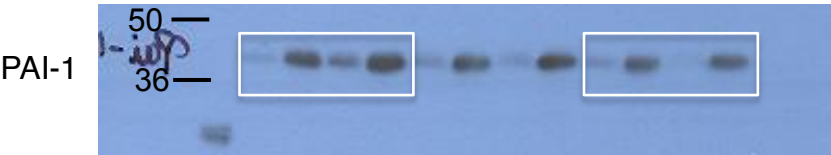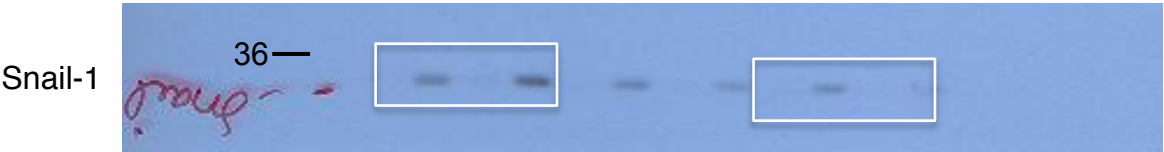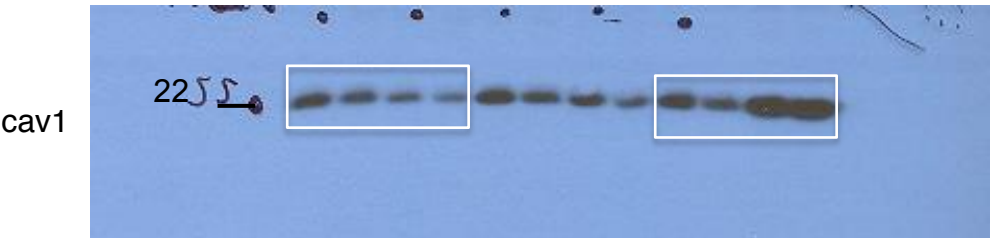

Supplement: Supplementary file 9 [file emmm0007-0102-sd9.pdf]

## Supplementary Figure 4

# B

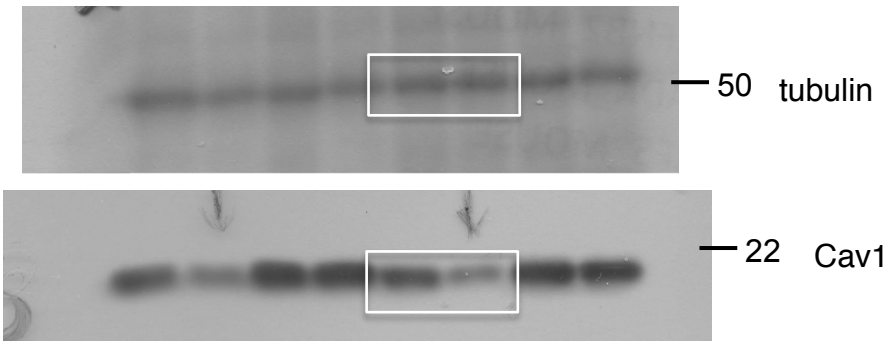

# C

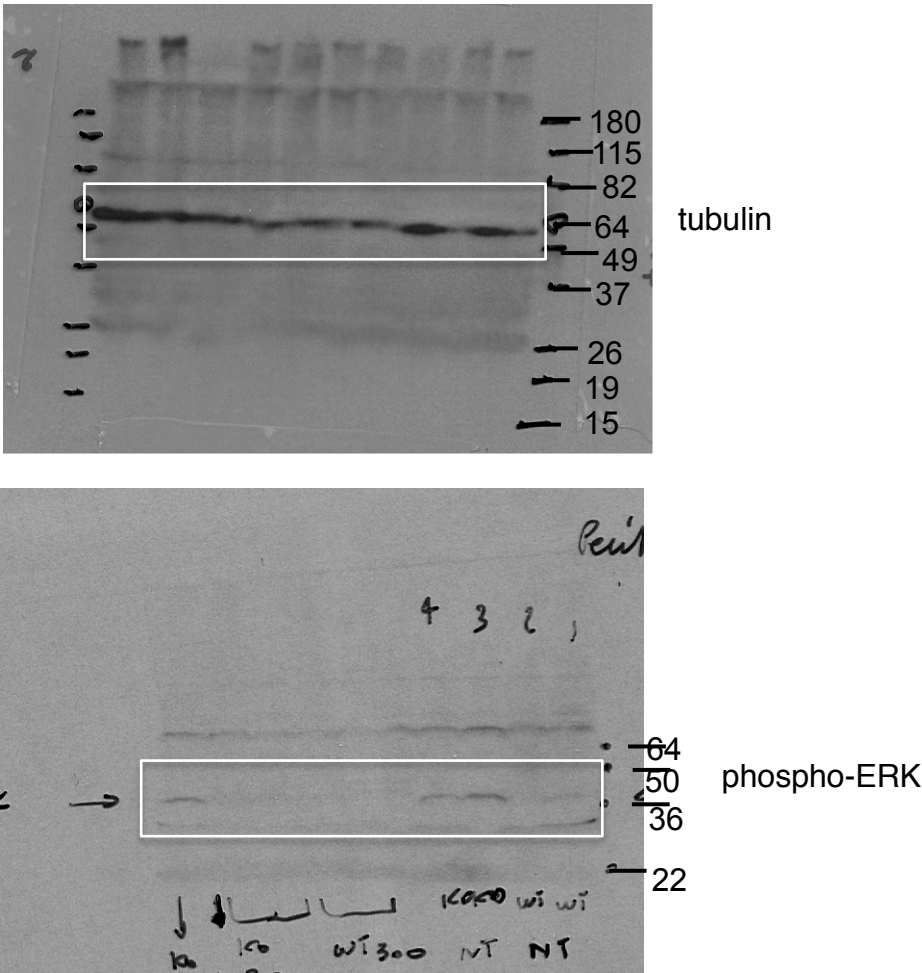

Supplement: Supplementary file 10 [file emmm0007-0102-sd10.pdf]

Supplementary figure 6

B

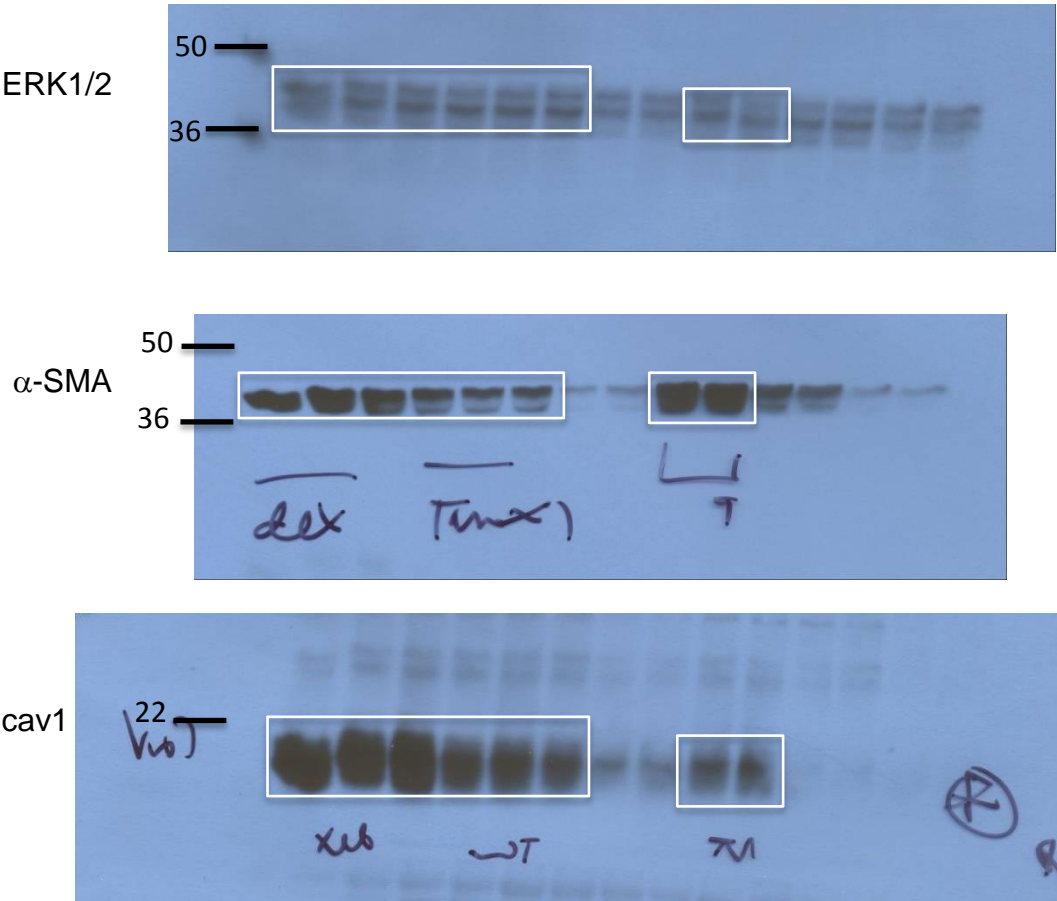

Supplement: Supplementary file 11 [file emmm0007-0102-sd11.pdf]

Figure 1

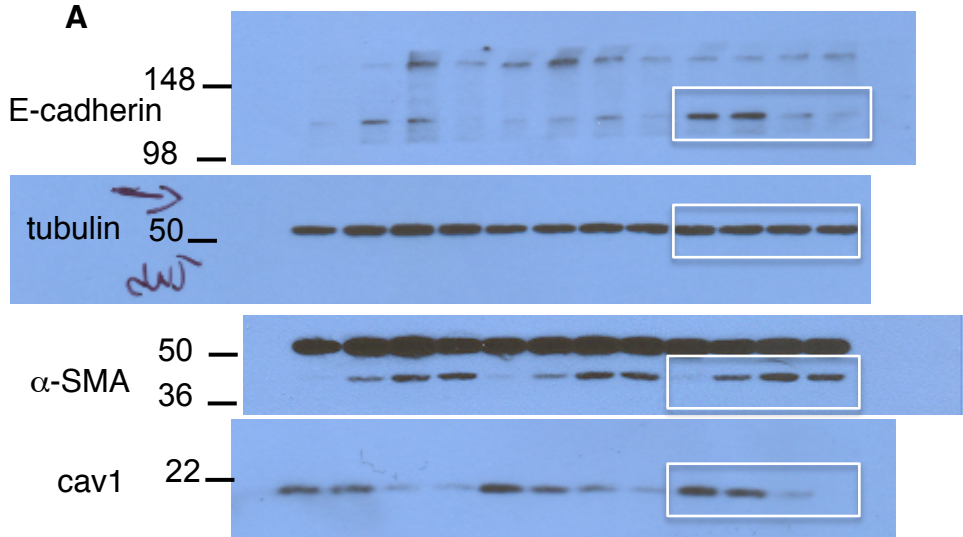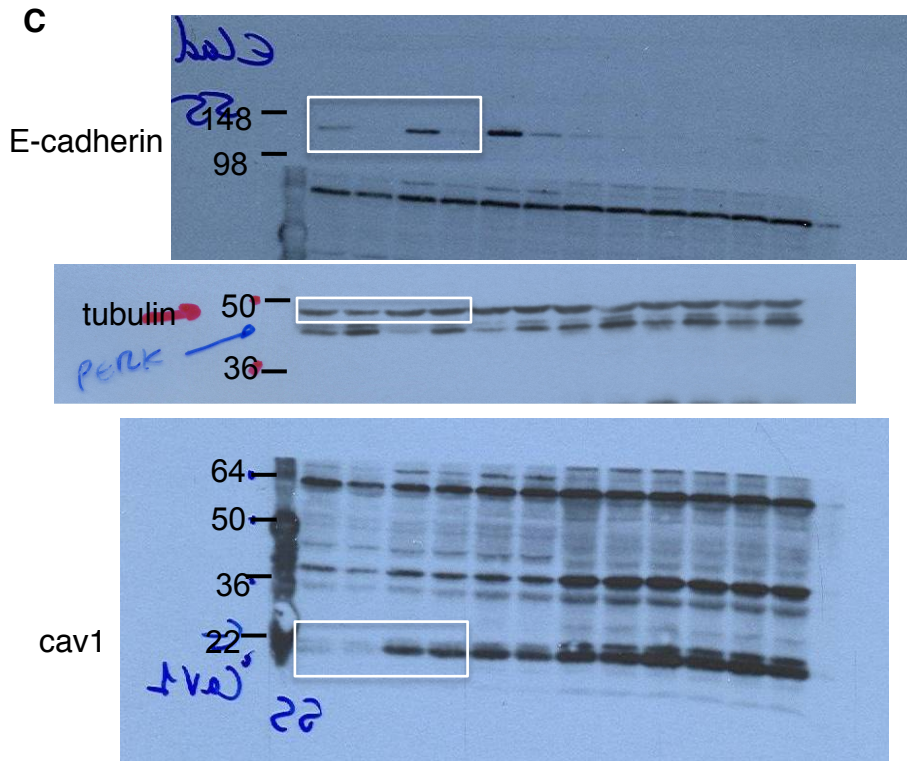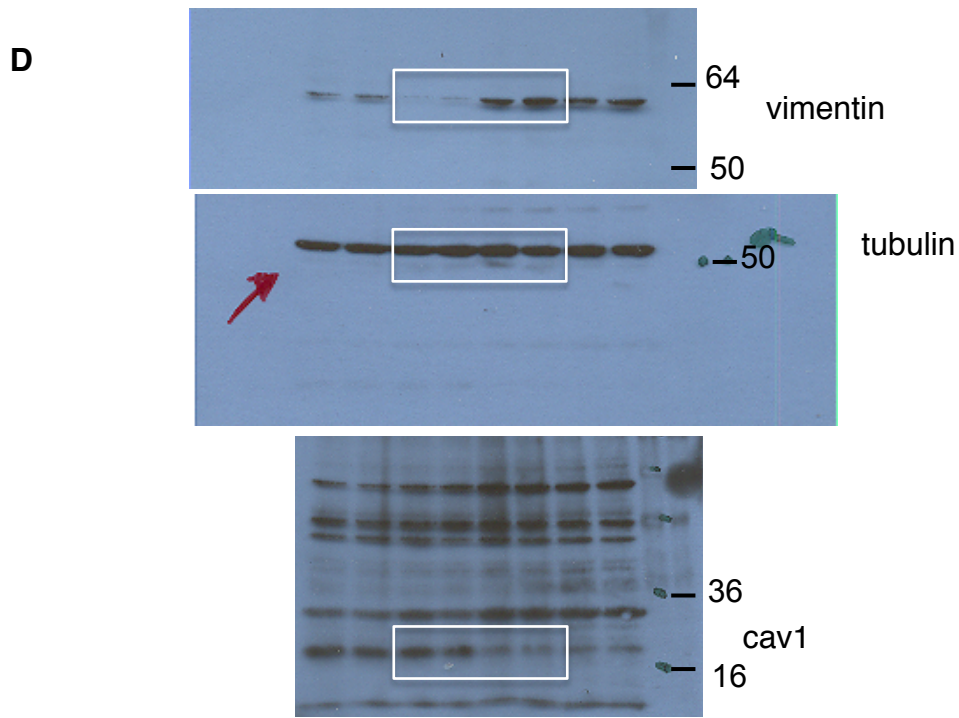

Supplement: Supplementary file 13 [file emmm0007-0102-sd13.pdf]

Figure 4

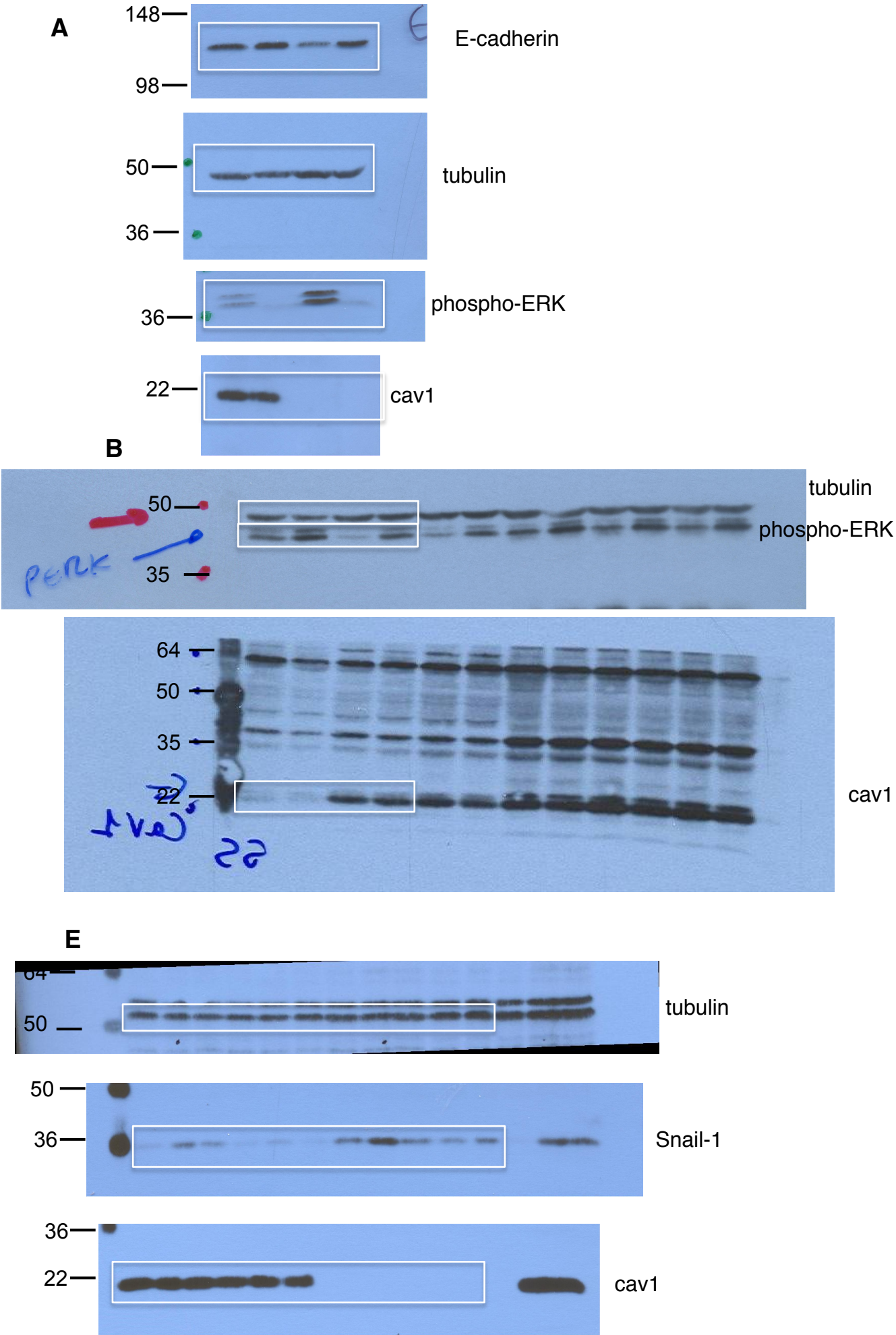

Supplement: Supplementary file 14 [file emmm0007-0102-sd14.pdf]

Figure 9

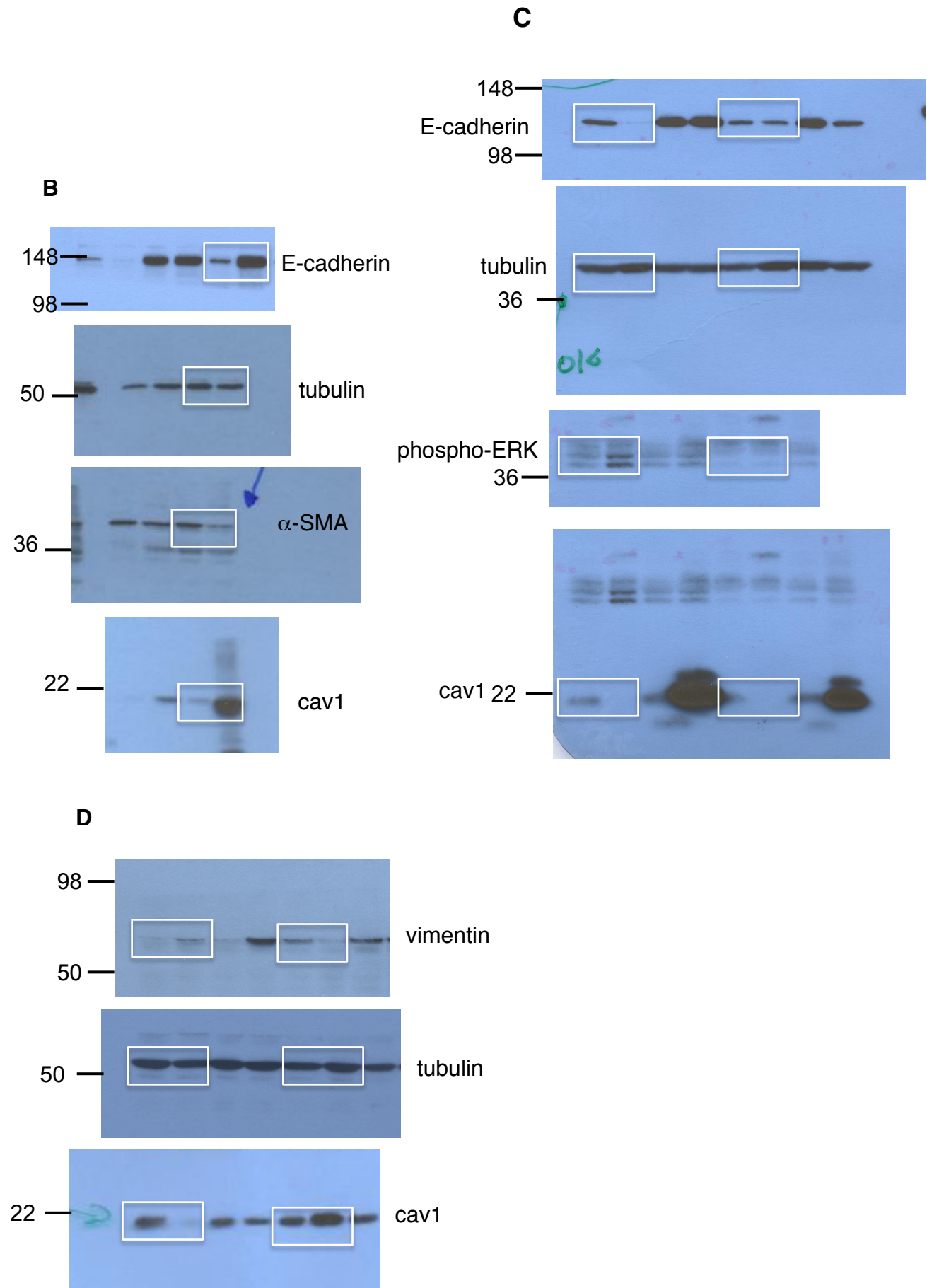

Supplement: Supplementary file 15 [file emmm0007-0102-sd15.pdf]
