# Supplementary material for: Caveolin-1 deficiency induces a MEK-ERK1/2-Snail-1-dependent epithelial–mesenchymal transition and fibrosis during peritoneal dialysis
Source: EMBO Mol Med. 2014 Dec 30;7(1):102–23. doi: 10.15252/emmm.201404127 (PMC4309670; doi:10.15252/emmm.201404127)
Supplement: Supplementary file 6 [file emmm0007-0102-sd6.pptx]

## Slide 1
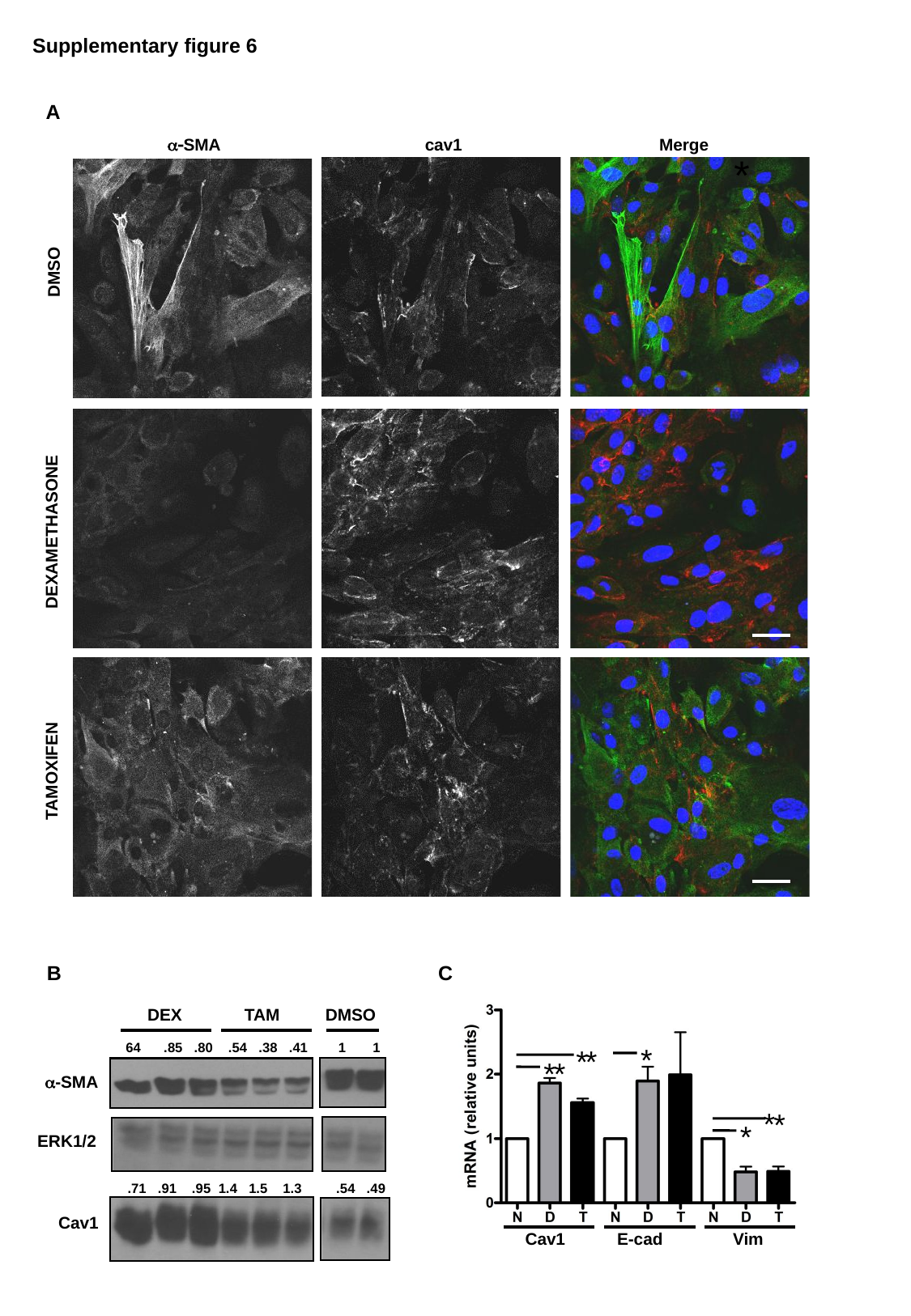

Supplementary figure 6
A
a-SMA
cav1
Merge
*
DMSO
DEXAMETHASONE
TAMOXIFEN
B
C
*
*
*
*
*
Cav1
E-cad
 Vim
TAM
DEX
DMSO
 64 .85 .80 .54 .38 .41 1 1
*
*
a-SMA
*
ERK1/2
 .71 .91 .95 1.4 1.5 1.3 .54 .49
Cav1
