# Supplementary material for: Caveolin-1 deficiency induces a MEK-ERK1/2-Snail-1-dependent epithelial–mesenchymal transition and fibrosis during peritoneal dialysis
Source: EMBO Mol Med. 2014 Dec 30;7(1):102–23. doi: 10.15252/emmm.201404127 (PMC4309670; doi:10.15252/emmm.201404127)
Supplement: Supplementary file 8 [file emmm0007-0102-sd8.docx]

**Supplementary figure legends**

**Supplementary figure 1 Effect of Cav1 targeting in expression and localization of proteins expressed at cell junctions. (A)** Quantification of elliptical factor of the experiment shown in **Fig. 1B**. *P* <0.001. (**B-F**) confocal IF of HPMCs treated as in **Fig. 1B**. Cells were fixed, permeabilized and stained with a monoclonal antibody against α catenin (**B**), β catenin (**C**), γ catenin (**D**), occludin (**E**), Cav1 (**F**). Scale bar: 15 μm. Inset: magnifications obtained through STED super-resolution microscopy. Representative images are shown of three experiments performed.

**Supplementary figure 2.** **Effect of ERK1/2 on the transcriptional activities and C-terminal phosphorylation of SMAD3 and SMAD1-5-8. (A)** Effect of pharmacologic inhibition of MEK on Snail and phospho-MEK1 expression in MCs from WT or Cav1^-/-^ mice. Confocal IF of MCs from WT (left) and Cav1^-/-^ mice (right). Cells were treated with 2 μM CI-1040 or DMSO for 12h, fixed, and immune- stained for phospho-MEK1 and Snail. Nuclei were stained with Hoechst 33342 (blue). Only CI-1040-treated cells are shown. See also Fig 4C. Scale bar: 30 μm. (**B-C**) MeT-5A cells were transiently transfected with the CAGA-luc reported plasmid to detect transcriptional activity of SMAD3 (*P* = 0.005) (**B**) and with BRE-luc reporter plasmid to detect transcriptional activity of SMAD1–5–8 .(*P* = 0.003) (**C**). Transfected cells were pretreated with DMSO or CI-1040 (2 µM) and then stimulated with TGF-β1 (1 ng/mL) for 9h. Data are presented as mean promoter activity (relative luciferase units). All experiments were performed in duplicate and three independent experiments (for SMAD3) and five experiments (for SMAD1-5-8) were performed. (**D**) Western blot for phospho-SMAD3 and phosphoSMAD1-5-8 in total HPMC lysates. Cells were treated with DMSO or CI-1040 (2 µM) for 1h and then stimulated with TGF-β1 (0.5 ng/ml) for 1h. Tubulin was detected as a loading control. Data are representative of three independent experiments. (**E**) Western blot showing expression of FN, PAI-1, Snail-1 and Cav1 in whole-cell lysates of HPMCs either transfected with control (scr) or Cav1-targeting siRNAs (siCav1) (left) or infected with control (L) or Cav1-expressing lentivirus (Cav1) (right). Data are representative of three independent experiments.

**Supplementary figure 3**

**(A)** Quantification of elliptical factor in experiments shown in **Fig 5A**. Bars represent means + s.e.m. of three independent experiments. P<0.001. n.s.: not significant. (**B-C**) Quantitative analysis of the experiment shown in **Fig. 5C.** Fluorescence intensity profiles of E-cadherin (B) and ZO-1 (C) in HPMCs trasfected with Cav1-targeting siRNA and treated with CI-1040. Fluorescence intensity profiles were analysed with Leica LAS-AF software and represented graphically with Prism Graph-Pad 5.0. In brief, lines were drawn from a few microns from the cell-to cell contacts towards the cell inside and fluorescence intensity was obtained (Fig 5B-C, left). 4 areas per cell and 10 couples of cells per condition were analysed, from two independent experiments. In the top right, total analysis is shown. The histograms in the bottom right refer to measure of fluorescence intensities within the bar in the image shown in the top. Scale bar: 10 μm. P<0.001.

**Supplementary figure 4. ERK1/2 proteins are phosphorylated in the parietal peritoneum of mice exposed to PD fluid and upon treatment with CI-1040**

(**A**) WB showing the expression of phospho-ERK1/2 and total ERK1/2 from parietal peritoneum of WT and Cav1^-/-^ mice exposed to PD fluid 5 times per week for three weeks (the experiment shown in Figure 8). The histogram shows the ratios of phospho-ERK1/2 and total ERK1/2 band intensities from seven WT and seven Cav1^-/-^ mice. P = 0.045. (B) WB showing the expression of Cav1 and tubulin in whole-cell lysates of MCs from WT mice exposed to PD fluid (diluted 1:1 in cell medium) for two days. Data are representative of three independent experiments. (**C**) Phospho-ERK1/2 and total ERK1/2 in parietal peritoneum of WT and Cav1^-/-^ mice treated with the indicated doses of CI-1040 or diluent for 6h.

**Supplementary figure 5. Exposure to PD fluid does not increase proliferation (Ki67 expression ) in the peritoneum of Cav1^-/-^ mice.**

Quantification of cell proliferation (Ki67-positive cells) in parietal peritoneal sections subjected to IF microscopy analysis in the experiment described in Fig. 8. n.s.: not significant

**Supplementary figure 6**

**Increased Cav1 expression upon treatment with dexamethasone and tamoxifen correlates with EMT reversal in HPMCs from PD patients.**

**(A)** Confocal IF of α−SMA (green), Cav1 (red) and Hoechst (blue) in HPMCs from the peritoneal effluent of patients undergoing PD. Cells were left untreated or treated for 48 hours with tamoxifen (4μM) or dexamethasone (1μM). Scale bar: 25 μm (**B**) Western blot for α−SMA and Cav1 in whole cell lysates of effluent-derived HPMCs treated as in (A). ERK1/2 expression was detected as a loading control. **(C)** Differential mRNA expression of Cav1, E-Cadherin and vimentin in HPMCs from PD patients treated as in (A). Quantitative RT-PCR was performed on total RNA. Histone H3 mRNA levels were used for normalization. Bars represent means ± SEM of duplicate determinations in four independent experiments. P = 0.002 and 0.0075 for Cav1; P = 0.027 for E-cadherin; P = 0.014 and 0.0097 for vimentin.

**Supplementary figure 7. Expression of ICAM1 in mouse MCs**

FACS analysis of mouse MCs. Mouse embryonic fibroblasts (MEFs) and MCs from WT and Cav1^-/-^ mice were detached, fixed, and stained with monoclonal anti-ICAM1, and analyzed by flow cytometry.
