# Supplementary material for: Caveolin-1 deficiency induces a MEK-ERK1/2-Snail-1-dependent epithelial–mesenchymal transition and fibrosis during peritoneal dialysis
Source: EMBO Mol Med. 2014 Dec 30;7(1):102–23. doi: 10.15252/emmm.201404127 (PMC4309670; doi:10.15252/emmm.201404127)
Supplement: Supplementary file 12 [file emmm0007-0102-sd12.pdf]

Caveolin-1 deficiency induces a MEK-ERK1/2-Snail-1-dependent epithelial-mesenchymal transition and fibrosis during peritoneal dialysis.

Raffaele Strippoli, Jesús Loureiro, Vanessa Moreno, Ignacio Benedicto, María Luisa Pérez Lozano, Olga Barreiro, Teijo Pellinen, Susana Minguet, Miguel Foronda, Maria Teresa Osteso, Enrique Calvo, Jesús Vázquez, Manuel López Cabrera, and Miguel Angel del Pozo

Corresponding author: Raffaele Strippoli, Centro Nacional de Investigaciones Cardiovasculares CNIC

Review timeline:

Submission date:	05 September 2013
Editorial Decision:	09 September 2013
Appeal:	11 September 2013
Editorial Decision:	16 September 2013
Additional author correspondence:	20 September 2013
Additional editorial correspondence :	23 September 2013
Resubmission:	02 April 2014
Editorial Decision:	30 April 2014
Appeal:	24 June 2014
Editorial Decision:	26 June 2014
Revision received:	23 October 2014
Editorial Decision	11 November 2014
2nd Revision	01 December 2014
Accepted:	05 December 2014

Transaction Report:

(Note: With the exception of the correction of typographical or spelling errors that could be a source of ambiguity, letters and reports are not edited. The original formatting of letters and referee reports may not be reflected in this compilation.)

Editor: Roberto Buccione

1st Editorial Decision 09 September 2013

Thank you for the submission of your manuscript "Caveolin-1 deficiency induces a MEK-ERK1/2-Snail-dependent epithelial-mesenchymal transition in the peritoneum".

I have now had the opportunity to carefully read your paper and the related literature and I have also discussed it with my colleagues. I am afraid that we concluded that the manuscript is not well suited for publication in EMBO Molecular Medicine and have therefore decided not to proceed with peer review.

You find that Cav1 knock-down induced EMT and hyperactivated Erk1/2 in human primary mesothelial cells (MCs) and that Cav1-/- mice featured MC changes consistent with the in vitro findings. You also find that in a mouse model of peritoneal dialysis (PD), the parietal peritoneum had a thicker submesothelial space in Cav1-/- mice, which was increased by exposure to PD fluids

and that ERK1/2 activation was higher in peritoneal tissue from Cav1-/- mice exposed to PD fluids compared to WT tissue. We appreciate that treatment with the MEK inhibitor CI-1040 during PD fluid exposure abolished ERK1/2 phosphorylation in WT and Cav1-/- mice and reduced peritoneal thickening and EMT and that in human MCs derived from patients undergoing PD, Cav1 expression inversely correlated with alphaSMA.

We acknowledge the potential interest of your findings on caveolin 1 and the observation that MEK inhibition might be beneficial for PD-related PM inflammation and injury. However, due to the well-documented connection between caveolin 1 and EMT, the prior knowledge that this can occur via a beta-catenin/GSK-3beta/Snail/TGFbeta/SMAD axis, and that it has been shown (as per your own work) that modulation of the ERK/NF-kB/Snail1 pathway may provide an avenue to counteract the structural and functional deterioration of the PM during PD, we are not persuaded that your manuscript provides the striking conceptual advance and the mechanistic novelty we would like to see in an EMBO Molecular Medicine article.

Appeal 11 September 2013

Thanks very much for your decision letter on our manuscript EMM-2013-03469 "Caveolin-1 deficiency induces a MEK-ERK1/2-Snail-dependent epithelial-mesenchymal transition in the peritoneum".

We would like to appeal your decision not to send the paper out for peer-review. Please find enclosed a formal appeal letter in which we expose several solid arguments to defend our claim.

We kindly hope you find these reasons appealing and change your initial decision.
Thanks very much for your consideration.

We would like to offer some comments regarding your decision to reject our manuscript EMM-2013-03469 entitled “Caveolin-1 deficiency induces a MEK-ERK1/2-Snail-dependent epithelial-mesenchymal transition in the peritoneum.”

While your editorial decision acknowledged *“the potential interest of your findings on caveolin 1 and the observation that MEK inhibition might be beneficial for PD-related PM inflammation and injury*”, our study was not selected for peer review on the basis of *“the well-documented connection between caveolin 1 and EMT”*, and the fact that we have previously provided insight into the role of the ERK/NF-kB/Snail1 axis in EMT progression.

We would like to raise the following points:

1. The connection between caveolin-1 and EMT is in fact poorly documented and far from being well understood. An old paper (Lu et al., Cancer Cell, 2003) linked *down-regulation* of caveolin-1 to E-cadherin downregulation and Snail induction. However, these results were obtained in vitro in cancer cell lines and with less reliable techniques (antisense-oligonucleotide). More recently, Bailey (JBC, 2008) linked caveolin-1 *upregulation* to EMT induction in tumors. Thus in different tumor models caveolin-1 can either block or induce EMT and tumor progression.
   A study published while our manuscript was in preparation (Li and Jimenez, Am J Pathol, 2013) associated absence of caveolin-1 with the induction of Endothelial to Mesenchymal transition (EndMT). This confirms our study on EndMT, but overlaps with our article in only part of 1 figure. Other studies linked absence of caveolin-1 with fibrosis, especially in the lung idiophatic fibrosis and in scleroderma, but the role and the onset of EMT in the pathogenesis of these complex diseases has never been demonstrated before. Our study introduces the concept that the absence of caveolin-1 drives a *complex* reprogramming of

mesothelial and endothelial cells towards a mesenchymal status, which is *not a simple increase of extracellular matrix protein production, as in the case of fibrosis*. Moreover, until this manuscript no studies had been conducted on the role of caveolin-1 in the reprogramming of peritoneal mesothelial cells, which are essential for the integrity of the peritoneal membrane and for the maintenance of the dialytic function. Thus, we feel that more research, as presented in our study, was needed in order to dissect the connection between caveolin-1 and EMT in different experimental systems and in different pathologies.

2. We demonstrated some years ago that the ERK/NF-kB/Snail1 axis plays a major role in the induction of EMT in peritoneal cells. However, these data were obtained through *in vitro* and *ex vivo* studies, not in an *in vivo* mouse peritoneal dialysis model, as in the current work. More importantly, our previous studies on ERK/NF-kB/Snail1 in EMT did not investigate a role for caveolin-1 at all, and cross-talk of MAPK with the SMADs was not analyzed. Through *in vivo* analysis and the silencing and ectopic re-expression of caveolin-1 in human primary cells, we now provide a major advance in the understanding of the interplay between caveolin-1, MEK pathways and EMT-fibrosis in a non-tumoral model of inflammation.

3. Last, our study is, to the best of our knowledge, the **first to perform peritoneal dialysis in genetically modified animals**. The use of mice for peritoneal dialysis studies promises to increase the understanding of molecular determinants responsible for the peritoneal damage associated with this form of replacement therapy and its subsequent withdrawal.

In the light of these comments, we kindly ask you to reconsider the rejection of our manuscript and send it out for peer-review.

Thank you again for your time and consideration.

2nd Editorial Decision 16 September 2013

Thank you for your message regarding our recent decision on your manuscript entitled "Caveolin-1 deficiency induces a MEK-ERK1/2-Snail-dependent epithelial-mesenchymal transition in the peritoneum".

I do understand your request to reconsider our initial decision and I thank you for highlighting the key aspects of the manuscript. However, please note that in our initial assessment, we had already considered the very same points you raise. Nevertheless, we have now thoroughly re-discussed your manuscript without prejudice.

We remain unconvinced that the connection between Caveolin 1 and EMT is novel enough, also considering, in addition to the papers you mention in your letter, recent reports by Lisanti's group (Cell Cycle, 10:3692, 2011) and by Huang et al. (Cancer Res 72: 655, 2012). We understand that you make further progress by extending these findings to a novel setting; nevertheless this previous knowledge limits the conceptual innovation of your study.

As mentioned in my first letter, we appreciate that you show that MEK inhibition might be beneficial for PD-related PM inflammation and injury, but we also feel that it derives directly from your previous work showing both the relevance of the ERK/NF-kB/Snail1 pathway and the importance of EMT in the deterioration of the peritoneal membrane during peritoneal dialysis. Indeed, our impression is that this particular observation does not appear to rely on, or require, the Cav 1-centred parts of the manuscript. Indeed, the MEK inhibitor appears to be efficacious both in wild-type and cav1-/- mice.

We do agree that your previously published PD model is a useful resource in understanding the pathological consequences of this form of interventional therapy. In this case however, we feel that

the translational implications at this time are limited to the MEK inhibitor effects mentioned above. As for the translational implications of your findings deriving form the potential role of Cav 1 in PD-related PM inflammation and injury, these remain to be developed by providing proof-of-principle experiments via drug-mediated modulation of Cav-1 levels in wild-type mice.

In conclusion and for the above-mentioned reasons, I am sorry to write that we have decided to uphold our original decision in not sending this manuscript out for in depth peer review. We do remain interested in your work, however, and if you could provide experiments based on drug-mediated modulation of caveolin and better integrate the MEK inhibitor findings with the caveolin-related aspects, I would commit to sending out the manuscript for review.

Again, I am sorry that I could not bring better news.

Additional author correspondence 20 September 2013

Thanks very much for the consideration of our work and for allowing us to resubmit a revised version including the new experiments you suggested.

We understand your feeling that the previously identified MEK-ERK1/2-Snail pathway is 'per se' sufficient to drive EMT and fibrosis of the peritoneum. We already have unpublished experiment and we guess that we can provide more evidence in order to support our hypothesis that Cav1 and MEK pathway are functionally connected in peritoneum and Cav1 may act as a main 'gate-keeper' of signalling events leading to inflammatory EMT.

Before defining a prospect of experiments that we can perform, I have a doubt regarding the interpretation of a sentence in your last message. You wrote: 'As for the translational implications of your findings deriving form the potential role of Cav 1 in PD-related PM inflammation and injury, these remain to be developed by providing proof-of-principle experiments via drug-mediated modulation of Cav-1 levels in wild-type mice.

Since both peritoneal dialysis fluid and MEK inhibitor may be considered as 'drugs', I would like to perfectly understand what do you mean exactly by "drug-mediated modulation' . My favorite interpretation is that a formal proof of the role of Cav1 in vivo (i.e. modulation of Cav1 levels in vivo by exposing the peritoneum to peritoneal dialysis fluids) is missing in our manuscript.

I am sorry to bother you but please could you clarify this key thing, so that we can prepare a convenient response letter and experimental plan.

Additional editorial correspondence 23 September 2013

Thank you for your message.

As I mentioned in my previous letter (with apologies if I did not make myself clear enough), we felt that two aspects are missing in the manuscript and that do not make it suitable at this stage for EMBO Mol Med:

1) an experimental in vivo setting to show whether direct modulation of Caveolin 1 levels has a therapeutic potential to affect PD-related PM inflammation and injury. I had mentioned "drug-mediated modulation" as we were intrigued by the mention of such an approach in your original cover letter.
2) solid causal connections between the MEK inhibitor findings and the caveolin-related aspects. You put it a bit more bluntly, but yes, proof of the role of Cav1 in vivo needs to be stronger.

Again, although it would be ultimately up to the Reviewers to decide, if you could provide further experimentation on the above, we would send out the manuscript for review.

You do not need to send me a response letter to this effect and could simply mention the key aspects that have been improved when and if you decide to re-submit to EMBO Mol Med.

Resubmission 02 April 2014

We have just submitted the revised version of our manuscript "Caveolin-1 deficiency induces a MEK-ERK1/2-Snail-dependent epithelial-mesenchymal transition in the peritoneum" (EMM-2013-03469-V2-Q). We greatly appreciate your suggestions to strengthen our manuscript before sending it to reviewers. According to them, the aim of our work during this time was to reinforce the link between Cav1 expression and ERK pathway in vivo, to provide evidence of 'drug mediated modulation' of Cav1, and to deepen some results already present in our manuscript.

We found that ex vivo treatment of mesothelial cells from WT mice with peritoneal dialysis (PD) fluid markedly reduced Cav1 expression (Supplemental Fig. 2B). These results demonstrate that similarly to TGF-b (Fig.1A), PD fluid reduces Cav1 expression in mesothelial cells and suggest that the same effect may occur in vivo. Unfortunately, we were unable to find clear changes of Cav1 expression in sections of peritoneum from WT mice exposed to peritoneal dialysis fluid. This is most likely due to a technical constraint, since Cav1 is strongly expressed in mesothelial monolayer in basal conditions, and hence relative changes of protein expression may be difficult to quantify in our experimental setup.

Moreover, we analysed whether Cav1 expression could be modulated by drugs already known for their anti-inflammatory/anti-fibrotic effects. To this purpose, we treated mesothelial cells from patients undergoing PD with dexametasone and tamoxifen. In these conditions, we found that increased expression of Cav1 triggered by these drugs correlated with reacquisition of epithelial-like features. We provide evidence by IF, WB and RT-PCR experiments (Supplemental figure 4). These results may be relevant in order to evaluate the 'therapeutic' potential of Cav1 modulation.

Last, and according also to your valuable advice, we were interested in expanding the results shown in Fig. 1F and 2A, i.e. the increased expression of extracellular matrix (ECM) proteins in peritoneum from Cav1-/- mice. We performed quantitative proteomics analysis of extracellular matrices derived from murine embryonic fibroblasts (MEFs) from Cav1 WT and Cav1-/- mice. We were not able to use murine mesothelial cells due to the small amount of ECM that can be obtained from primary cultures of these cells. However, fibroblasts and mesothelial cells cooperate in production of ECM in normal and pathologic conditions in the peritoneum, supporting our approach. Also, it should be noted that MEFs are still very undifferentiated cells conserving endo-, meso- and ecto-dermal features. We found that MEFs from Cav1-/- mice produce increased amount of ECM proteins, such as collagens, fibronectin and laminin. Also, we found evidence of increased expression of proteins related to TGF-b activation such as Latent-transforming growth factor beta-binding protein 2, SPARC, Thrombospondin-1 and serpins. These results confirm and integrate other results shown in our manuscript (Fig. 3 and Supplemental Fig. 1), where we demonstrate a hyperactivation of TGF-b mediated pathways (SMAD2/3, ERK1/2) in mesothelial cells from Cav1-/- mice and in human cells where Cav1 was silenced. Therefore, the quantitative proteomics approach strengthens the link between Cav1 expression and ERK pathway and integrates global changes in the ECM proteome. This approach was time-consuming and hence delayed significantly the resubmission of the paper, but we think that the results underline significantly our conclusions.

In summary, we believe that we have substantially improved the quality of our manuscript, and we hope that you find now it suitable to progress through peer revision in EMBO Molecular Medicine.

3rd Editorial Decision 30 April 2014

Thank you for the submission of your manuscript to EMBO Molecular Medicine. We have now heard back from the three Reviewers whom we asked to evaluate your manuscript.

You will see that while one Reviewer is supportive of your work the other two are less so with varying degrees of concern. Considered in aggregate, the concerns expressed prevent us from considering publication. I will not dwell into much detail, and just mention some fundamental points that I wish to bring to your attention.

Reviewer 1 offers little commentary but is clearly positive. I do note however that s/he emphasises the effects of MEK inhibition on peritoneal fibrosis, which we agree is an interesting aspect of the work that remains however disconnected from the caveolin aspects.

Reviewer 2 is clearly negative and directly challenges the conceptual novelty and mechanist insight provided by the study.

Reviewer 3, while clearly more supportive, raises a number of technical concerns, but more importantly, mentions two important shortcomings which essentially overlap with Reviewer 2's concerns. On one hand the findings are considered to be too descriptive and on the other that the manuscript essentially delivers a message which is not novel, although applied to a novel setting.

Although we remain appreciative, as do the Reviewers, of the quality of your work and we recognize its potential interest, due however to these fundamental concerns and the overall lack of enthusiasm by the critical Reviewers, and after extensive discussion with my colleagues, I am returning the manuscript to you at this stage so that you may rapidly seek publication elsewhere.

I am sorry that I could not bring better news.

***** Reviewer's comments *****

Referee #1 (Remarks):

Peritoneal dialysis is a good choice of renal replacement therapy particularly for patients at their onset of dialysis history. Unfortunately, with ongoing years in many cases the method cannot be performed anymore due to peritoneal fibrosis. A number of mechanisms play a role in that process, EMT being one of them. In the current manuscript Strippoli and colleagues are to be applauded for pointing to a central role of Caveolin-1 in that process. Using Cav1-/- mice they were able to Show that These mice developed EMT driven peritoneal fibrosis. Preventing MEK resulted in reversal of EMT and peritoneal fibrosis. The studies are well performed technically and convincing. In the western blots I would have added the size of the bands. Other than that I have no objections to this classy work.

Referee #2 (Comments on Novelty/Model System):

As outlined in the comments to the authors, there is not much conceptual or mechanistic novelty with the findings presented here.

Referee #2 (Remarks):

This paper by Strippoli and colleagues shows compelling evidence that Cav-1 plays a role in mesothelial cell EMT. EMT is triggered by exposure of MCs to hyperglycemic and hyper osmotic dialysis fluids or TGFbeta.

General comments: This is a straightforward manuscript. The experiments are well-designed, well-executed and the conclusions are justified based on the data presented. The major concern is the relative impact of the findings, that may justify publication in a more specialized journal. Caveolin and its impact on EMT, as well as on downstream signaling through the Erk pathway has been

widely studied for more than a decade. While the setting may be novel in the context of peritoneal dialysis, the basic mechanisms at hand are not new and all have previously described on multiple occasions. One of the recent examples (from many) includes Cell Cycle. 2011;10(21):3692-700 that deals with this issue of EMT, caveolin and TGFbeta signaling. The findings here are incremental, and do not provide any new mechanistic insights.

Minor Issues:
A number of small typos throughout the paper, such as on page 6 (60-60 nm diameter ... treatement).

Biogro-2 is added to the MC media. It contains a number of growth factors, including insulin. What are the consequences of that?

Referee #3 (Remarks):

The manuscript submitted by Strippoli et al. addresses an interesting medical condition pertaining to peritoneal dialysis. The authors have explored the role of caveolin in the control of mesothelial cell plasticity and permeability and in the induction of fibrosis. Previous studies from this laboratory have provided evidence that mesothelial cells can undergo an Epithelial-Mesenchymal Transition (EMT) through the TGF non-canonical pathway and implicated Snail-1 as a main driver of this morphological and biochemical conversion. Several laboratories, including the authors' laboratory, have studied in some depth the role of caveolin in the control of cell plasticity and permeability. The current study, focusing on mesothelial cells, delivers a similar message.

Criticisms

Although most experiments were conducted quite rigorously, it is surprising to note that the adhesive properties of mesothelial cells have not been documented properly. For instance, E-cadherin immunolocalization is never shown throughout this study. Likewise, the expression pattern of other components of the adherens junctions, such as -catenin, -catenin and P120-catenin, should have been characterized. In addition, it would be critical to show whether desmosomes are altered through EMT in conjunction with the cytokeratin network reorganization. Finally, the status of tight junctions should be examined. Only JAM-A, an accessory protein of the zonula occludens, is analyzed in Figure 2C. The authors should examine the distribution of claudins, occludins and ZO-1.

Confocal images should be provided to estimate the degree of polarity of these cells. This is an essential point to be made in this study in order to understand how caveolin-1 affects apico-basal polarity.

Figure 1A shows that E-cadherin is almost completely down-regulated by TGF ; this down-regulation is significantly less in caveolin-1-null cells (Fig. 1E). Figure 1G is not convincing: There is likely a non-homogenous ECM density in different fields.

In Figure 2B, JAM-A is expressed in the caveolin-1-null cells: This is in conflict with the EMT process, knowing that tight junctions are the first to disappear.

Figure 3F-3G are of low quality; the immunodetection of Snail1, phospho-MEK and Smad2/3 are difficult to interpret.

In Figure 4, the immunolocalization of cytokeratin in mesothelial cells does not indicate a robust epithelial morphology. Similarly, cells treated with the MEK inhibitor, although exhibiting a more cuboidal morphology, do not show obvious cell-cell contact. Furthermore, TGF treatment does not seem to revert significantly the phenotype of cells treated with the MEK inhibitor.

The right micrograph is unable to be read in Figure 5C.

In Figure 7F, the data are not very convincing despite being one of the crucial figures of the study.

Figure 8A is also of very low quality; it seems that caveolin-1 localization pattern has a fibrillar pattern.

The model proposed in Figure 8 is very rudimentary and does not go beyond what has been published previously.

Overall, this study needs to be much better documented and should attempt to provide more than a phenomenological description of EMT and address, in greater depth, the role of caveolae with respect to TGF signaling.

Minor remarks
Referencing should be proof-read.
Snail should be changed to Snail-1 throughout.

APPEAL 24 June 2014

Thanks very much for the thorough review of our manuscript by both the referees and yourself. While the quality of our manuscript is generally appreciated, concerns are raised by Reviewers 2 & 3 about the novelty of the message but, as you underline in your letter, with varying degrees of concern. In fact, while Reviewer 2 expresses mainly negative concerns, Reviewer 3 is clearly more supportive, including in her/his comment about novelty (see below). Reviewer 1 is extremely positive ("Strippoli and colleagues are to be applauded…"). In fact we feel flattered by the praises to our work raised by this referee, since receiving such enthusiastic review is very uncommon nowadays. But Reviewer 3 also offers positive comments, and asks for specific experiments, that we could address if given the opportunity.

In light of the reviewer's comments, we would like to discuss with you about the two main concerns that currently preclude our manuscript to progress throughout the revision process, i.e.

1. Disconnection between Cav1 and ERK pathway in our study.

Regarding the first point, the absence of Cav1 has been associated to hyperactivation of the ERK pathway in many studies from many different groups (Ariotti et al, 2014; Burgermeister et al, 2011; Cerezo et al, 2009; Engelman et al, 1998). Indeed one of the first mechanistic studies about Cav1 was the description by several groups that many different oncogenes (Ras, v-Src, BCR-Abl, etc) decrease the expression of Cav1 via ERK pathway (Engelman et al, 1999). Conversely, cells from different tissues show ERK1/2 hyperactivation when Cav1 expression is silenced (Gosens et al, 2006; Murata et al, 2007; Tourkina et al, 2010). Therefore, ***all three*** reviewers accepted that block of ERK pathway is mechanistically connected with rescue of the pro-EMT Cav1-/- phenotype. None of them required additional experimental approaches to confirm the inverse correlation between Cav1 and ERK activity, including Referee 1 (here we disagree with your interpretation -see below-). In fact, one of the main arguments of Referee 2 to question the novelty of our study is that *"Caveolin and its impact on (...) downstream signaling through the Erk pathway has been widely studied for more than a decade. While the setting may be novel in the context of peritoneal dialysis, the basic mechanisms at hand are not new and all have previously described on multiple occasions"*. While we disagree with this reviewer in that *all* the mechanisms described are not new (see below on these other points), we completely agree that the ERK and Cav1 connection is so well established in the field that there was no point in further dwelling with it. In fact, this was the starting point of our study.

2. General novelty of our message.

The lack of novelty is claimed mainly by Reviewer 2, and to a lesser extent by Reviewer 3, whereas the Reviewer 1 is clearly positive.

A critical review of the literature on the subject of this study (the link between Cav1 and EMT in the mesothelium, with implications –acting on the ERK pathway- for prevention/therapy of peritoneal dialysis-induced fibrosis) actually demonstrates that many of the results we found and discuss were far from being established before this paper. In fact, Reviewer 1 states this:

"Peritoneal dialysis is (...). Unfortunately, with ongoing years in many cases the method cannot be performed anymore due to peritoneal fibrosis. A number of mechanisms play a role in that process, EMT being one of them. In the current manuscript Strippoli and colleagues ***are to be applauded for pointing to a central role of Caveolin-1 in that process****."*

In the same direction, Reviewer 3 claims that *"the manuscript delivers a message about the role of caveolin in the control of cell plasticity and permeability, which is similar to previous studies, but applied to a* ***novel setting (mesothelial cells)****"*.

Although we give more details in the response to this specific comment by Reviewer 2 (please see below), we summarize here that the Cav1/EMT link has been analyzed in a limited number of studies, and all of them have been conducted on tumor cell lines. Differently from untransformed cells (i.e. our study), in many tumors Cav1 promotes, rather than inhibits, EMT and invasion. On the other hand, when looking to the 'translational relevance' of our study, the analysis of peritoneal dialysis in Cav1$^{-/-}$ (i.e. the role of Cav1 in peritoneal EMT and fibrosis) mice is totally new. Also, only a few studies have dealt to date on the antifibrotic properties of MEK inhibitors, which are up to now increasingly studied mainly as antitumoral targets, but according to our novel findings, they may be relevant also for the treatment of non tumoral fibrotic diseases.

Based on these comments and on the literature, we strongly believe that our study is not limited to merely put forward a pre-acquired concept in a new experimental setting.

* * * * * * *

Apart from these two main concerns, Reviewer 3 raises reasonable criticisms, which we offer to experimentally address. We estimate that we will be successful in this goal, which will potentially render two positive referees out of three. Based on this reasoning, we kindly ask the Editor to reconsider the rejection of this manuscript and to allow us to improve the quality of our study by performing the experiments suggested by Reviewer 3, as well as by addressing the Cav1/Erk link and the novelty issues raised. We hope to significantly enhance the enthusiasm of Reviewer 3 addressing experimentally her/his claims, and we discuss that some of the conclusions of Reviewer 2 (those leading her/him to dampen the novelty of our study) may be challenged when the literature is carefully revised. With Reviewer 1 being already so positive and Reviewer 3 expressing mixed comments at the moment, we strongly feel that we should be given at least the chance to resubmit our study for further revision.

4th Editorial Decision 26 June 2014

I have now read your rebuttal letters and your proposed plan for revision. If you send us a rmanuscript revised as outlined in your letter, I will promptly send it out for re-review.

I look forward to seeing a revised form of your manuscript as soon as possible.

1st Revision - authors' response 23 October 2014

Point-by-point Response to Reviewer's comments

Referee #1

Strippoli and colleagues are to be applauded for pointing to a central role of Caveolin-1 in that process [i.e. EMT driven peritoneal fibrosis]
We thank Referee 1 for the extremely positive evaluation of the importance and the novelty of our findings.

Referee #2

As outlined in the comments to the authors, there is not much conceptual or mechanistic novelty with the findings presented here.
This paper by Strippoli and colleagues shows compelling evidence that Cav-1 plays a role in mesothelial cell EMT. EMT is triggered by exposure of MCs to hyperglycemic and hyper osmotic dialysis fluids or TGFbeta.

General comments: This is a straightforward manuscript. The experiments are well-designed, well-executed and the conclusions are justified based on the data presented. The major concern is the relative impact of the findings, that may justify publication in a more specialized journal. Caveolin and its impact on EMT, as well as on downstream signaling through the Erk pathway has been widely studied for more than a decade. While the setting may be novel in the context of peritoneal dialysis, the basic mechanisms at hand are not new and all have previously described on multiple occasions. One of the recent examples (from many) includes Cell Cycle. 2011;10(21):3692-700 that deals with this issue of EMT, caveolin and TGFbeta signaling. The findings here are incremental, and do not provide any new mechanistic insights.

We acknowledge Reviewer 2's appreciation to our approach and the way the study has been performed, while we respectfully disagree about the lack of novelty in the link between Cav1 and EMT. After the seminal contribution by Tony Hunter´s lab (Lu et al, 2003), this topic has received little attention. Most studies, including the Tony Hunter paper, deal with EMT in tumors. Therefore our paper is one of the few studies to implicate Cav1 in a non-tumor EMT experimental model (as noted by Reviewers 1 and 3). This is a key point since tumor transformation can dramatically modify the role of Cav1 (Pinilla et al, 2006). Therefore it is of extreme importance in the field to determine the relationship between Cav1 and EMT in non-tumor pathophysiology. For instance, the study cited by Reviewer 2 (Salem et al, 2011) is interesting and strongly supports our results. However, this study was performed entirely in just one pancreatic cancer cell line (PANC10), in which Cav1 was ectopically expressed. No experiments are shown in which Cav1 expression is reduced, and neither were $Cav1^{-/-}$ mice used in this study. Moreover, the analysis of signaling pathways involved is limited to just one figure. Thus, the information that can be obtained from this study about the role of Cav1 in EMT is far from being definitive, especially for a non-tumor setting.

Furthermore, and contrary to the novel findings of our paper, many studies, especially in breast cancer, demonstrated that *increased* Cav1 expression promotes EMT and tumor invasion. In fact, in triple negative (basal like) breast cancer (the most aggressive subtype) *increased* Cav1 expression is the marker that best correlates with EMT, even better than loss of E-cadherin (Hollestelle et al, 2013; Pinilla et al, 2006). In less differentiated tumors, Cav1 often *promotes* EMT and invasion. In striking contrast, in a non-tumor environment, we report **completely opposite** observations for Cav1 in mesothelial cells in a pro-inflammatory setting, highlighting the novelty of our findings and their importance in physiological models.

Thus, the link between Cav1 and EMT is far from being established in non-tumor diseases, and, after reviewing the literature, we can conclude that our results are novel.

Furthermore, regarding the 'translational relevance' of our study, the analysis of peritoneal dialysis in $Cav1^{-/-}$ mice (to examine the role of Cav1 in peritoneal EMT and fibrosis) is completely new. To our knowledge, this is the first time that peritoneal dialysis has been performed in genetically modified animals. Our study thus opens the way to further studies aimed at understanding the mechanism of EMT/fibrosis during peritoneal dialysis *in vivo*. Also, only a few studies to date have dealt with the antifibrotic properties of MEK inhibitors, which have mostly been studied as antitumoral targets, but which according to our findings might also be relevant to the treatment of non tumoral fibrotic diseases.

Based on these observations, we strongly believe that our study is not limited to merely putting forward a pre-acquired concept in a new experimental setting.

Regarding the mechanism involved in EMT induction, we acknowledge that our study does not identify a new molecule or signaling pathway. Rather, our study offers an integrated view of several pathways that all play a role in EMT induction: MEK-ERK, SMAD2-3/SMAD1-5, Snail-1 and their regulation by Cav1. This is explained in the updated final model (Figure 9). We strongly believe that our study is not 'devoid of mechanistic novelty' or simply 'incremental'. Through the analysis of mutual regulation of many pathways in a specific cell type and pathophysiological condition relevant to human health, we believe that our study provides advances in the understanding of basic mechanisms of EMT induction.

Minor Issues:
A number of small typos throughout the paper, such as on page 6 (60-60 nm diameter ... treatement).
We have corrected these and other typos.

© EMBO

Biogro-2 is added to the MC media. It contains a number of growth factors, including insulin. What are the consequences of that?
As we now state in the manuscript, Biogro-2 is routinely added to favor *in vitro* mesothelial cell differentiation, being washed out the day before performing the experiment.

Referee #3

The manuscript submitted by Strippoli et al. addresses an interesting medical condition pertaining to peritoneal dialysis (...)

We appreciate the positive comments of this reviewer about the relevance of the clinical issue we address. Regarding the novelty of this study, we refer the Reviewer to our response to Reviewer 2.

(...) E-cadherin immunolocalization is never shown throughout this study.
We thank the reviewer for this observation, which prompted us to analyze this important issue further. We did not show E-cadherin immunolocalization in the previous version of our study, and to date the literature lacks studies analyzing E-cadherin localization in mesothelial cells using newly developed imaging technologies. Expression of E-cadherin and junction-associated molecules and their localization in the peritoneum deserves deeper study, especially considering the particular nature of mesothelial cells, which co-express epithelial and mesenchymal markers.
We now provide images showing E-cadherin staining in mesothelial cells using a new generation confocal microscope. The results are shown in new **Figures 1B** and **4B**. In untreated cells, E-cadherin is clearly localized at cellular junctions. Interestingly, Cav1 silencing reduces E-cadherin expression and completely subverts E-cadherin distribution at junctions, which is rescued upon treatment with MEK inhibitor CI-1040 (new **Figure 4B and supplementary figure 3B for quantification).**

These new data reinforce our previous conclusions. We found that Cav1 silencing profoundly disrupts the architecture of intercellular junctions, which is compatible with an EMT-like status in MCs in the absence of Cav1.

Alpha- and Beta-catenin,p120-catenin should be examined. (...)The authors should examine the distribution of claudins, occludins and ZO-1.

Besides E-cadherin, we extended our analysis to other intercellular junction proteins. We analyzed by confocal microscopy the localization of a-, b-, g-catenin, occludin and ZO-1. The results are shown in new **Supplementary Figure 1B and Figure 4B, bottom**. As with E-cadherin, the distribution of these junction proteins is profoundly altered by Cav1 silencing. Basically, these proteins disappear from the junctions. In the case of occludin and ZO-1, total protein levels are unchanged or increased upon Cav1 silencing. Also similar to the situation with E-cadherin, ZO-1 is re-acquired at cellular junctions upon treatment with CI-1040 (**Figure 4B and supplementary figure 3C for a quantification).**

Confocal images should be provided to estimate the degree of polarity of these cells. This is an essential point to be made in this study in order to understand how caveolin-1 affects apico-basal polarity.

The referee is absolutely right. In the previous version, we only analyzed the elliptical factor (i.e. the longest diameter of the cell divided by the shortest one, which is a measure of the degree of x-y cell polarity). We found that, in the absence of Cav1, cells acquire a spindle-like morphology **(Fig 1B, 4B** and **6E)**. We now clearly show that lack of Cav1 leads to disappearance of E-cadherin and ZO-1 from cell junctions (new **Fig. 4B-C**). Moreover, new 3D reconstruction experiments show that $Cav1^{-/-}$ cells have a flattened morphology, with reduced cell height (new **Fig 4B**). All these observations suggest a loss of apico-basal polarity and acquisition of a mesenchymal-like phenotype in the absence of Cav1. Interestingly, treatment with MEK inhibitor leads to a significant rescue of morphological and biochemical abnormalities in these cells (**new Fig4C**).

Figure 1A shows that E-cadherin is almost completely down-regulated by TGFβ this down-regulation is significantly less in caveolin-1-null cells (Fig. 1E).

The reviewer is right. E-cadherin is almost totally down-regulated upon treatment with TGF-b1 in human MCs, whereas there is a less intense, although still evident, reduction in murine MCs. In order to exclude a species bias (all the experiments were conducted with human recombinant TGF-b1) we have now performed experiments using murine TGF-b1. The results are shown in **Figure R1 (fig.1 for reviewer's inspection)**. We did not find any notable difference between results obtained with human or murine TGF-b1. Rather, based on this and other unpublished observations, we found a more stable phenotype in murine MCs.

Figure 1G is not convincing: There is likely a non-homogenous ECM density in different fields

Extracellular fibronectin accumulates in three-dimensional bundles, so on confocal microscopy part of the protein is often out of focus. The result shown in this image (increased FN expression in cells from Cav1$^{-/-}$ mice) is supported by RT-PCR experiments (**Fig1F**) and by the proteomics analysis of CDMs (cell derived matrices, new **Table1**). We have now applied a novel algorithm developed by co-authors Enrique Calvo and Jesús Vázquez, who are experts in Proteomics and Bioinformatics for systems-biology approaches. We have re-analyzed the data and now show that collagens, FN and other ECM proteins are generally more abundant in matrices from Cav1$^{-/-}$ cells (**new Table 1**) This reinforces the link between the absence of Cav1 and increased deposition of FN and TGFb-related ECM proteins.

In Figure 2B, JAM-A is expressed in the caveolin-1-null cells: This is in conflict with the EMT process, knowing that tight junctions are the first to disappear.

This comment is interesting. We described as 'punctate staining' the JAM-A expression in the peritoneum of Cav1$^{-/-}$ mice. We propose that cells from Cav1$^{-/-}$ pertoneum are in a 'pre-EMT' stage. The junctions are partly maintained, and the functionality of the organs is conserved, although we found increased basal permeability both in peritoneum and in vessels. Based on the experiment shown in **Figure R1** (E-cadherin downregulation upon treatment with murine TGF-b1) and other unpublished observations from our laboratory, we believe that murine MCs are less plastic (less prone to EMT induction) than their human counterparts, probably due to the establishment of homeostatic mechanisms in genetically modified mice.

the immunolocalization of cytokeratin in mesothelial cells does not indicate a robust epithelial morphology.

As already accepted in the literature, mesothelial cells express high levels of cytokeratin. This and other markers allow them to be easily distinguished from endothelial cells, macrophages and fibroblasts (Aroeira et al, 2007). We have already shown that peritoneal cells have an intense cytokeratin expression (Strippoli et al., 2008, Strippoli et al., 2010).
Cytokeratin expression is much more stable than other epithelial markers, and tends to persist even when MCs have already undergone EMT (Strippoli et al, 2012), FigS3.

Figure 3F-3G are of low quality; the immunodetection of Snail1, phospho-MEK and Smad2/3 are difficult to interpret.

We chose to show many nuclei in the same figure, in addition to the quantified data, in order to allow reviewers and readers to form a personal impression. To make our findings easier to interpret, we now provide images with increased magnification (new **Figure 3F-G)**.

Similarly, cells treated with the MEK inhibitor, although exhibiting a more cuboidal morphology, do not show obvious cell-cell contact.

In new **Figure 4A,** information about cell-cell contacts is provided by actin staining. Actin staining is widely used to obtain quantitative data on cell shape such as the elliptical factor, but may not be a good readout for the analysis of cell-cell contacts. Depending of the section chosen, actin may or may not to reach the cell contacts. To show more clearly the role of Cav1 and the effect of MEK inhibitors on cell-cell contact, we have now stained for E-cadherin, ZO-1 and actin in new **Figure**

4B-C (**and supplementary figure 3B for a quantification**). The results show that upon treatment with CI-1040, both E-cadherin and ZO-1 are re-expressed at cell contacts.

In Figure 7F, the data are not very convincing despite being one of the crucial figures of the study.

We apologize for the lack of clarity in the way we presented this critical figure. We agree with Reviewer 3 that these results are crucial, since they connect the effect of Cav1 absence in peritoneal EMT/fibrosis to a clinical parameter, thus amplifying the translational potential of our study. The ultrafiltration test shown in Fig. 7F demonstrates that peritoneum of Cav1$^{-/-}$ mice treated with PD fluid has less ultrafiltration capacity than that of WT mice, with a p value of 0.0023 (Mann–Whitney test). The reduced ultrafiltration capacity is associated with the more altered peritoneal architecture in the PD fluid-treated Cav1$^{-/-}$ mice. Moreover, MEK inhibition in this setting induces a significant recovery of the ultrafiltration defect in Cav1$^{-/-}$, with a p value of 0.0043. We understand Reviewer's interpretation, because the way we showed the data could mislead one into thinking that this was not the right comparison to make. This was because the connecting lines were not perfectly drawn; they now are. We have now labeled this more clearly and show the actual p values, which unquestionably show statistical significance **(Figure R2, corresponding to Fig. 7F**).

Figure 8A is also of very low quality; it seems that caveolin-1 localization pattern has a fibrillar pattern.

This is an interesting point about caveolin biology that also has been worked out by several labs, including ours. Different from clathrin, Cav1 was first described as having a 'trabecular staining pattern'. The group of Dick Anderson first published the cloning of Cav1 and the first anti-serum raised against this protein gave a trabeculated, fibrillar pattern, suggesting that caveolae might be linked to actin stress fibers (Rothberg et al, 1992). Then Van Deurs´s group showed that Filamin A provides a molecular link between Cav1 and actin. Van Deurs and Rob Parton demonstrated an electron-dense connection between caveolae and actin stress fibres by EM Tomography (Richter et al, 2008). Then, our group showed that caveolae co-align with actin stress fibers (Muriel et al, 2011) and that this co-alignment is required for caveolae trafficking, internalization and function (Echarri et al, 2012). Therefore we can say that the staining shown in Fig.8A is a typical caveolin staining for cells of myofibroblastic phenotype

The model proposed in Figure 8 is very rudimentary and does not go beyond what has been published previously.

We agree that our model was quite 'rudimentary' (or oversimplified), considering the vast amount of possible interconnections between Cav1, signaling pathways and master genes of EMT. However, to the best of our knowledge, no models are available in the literature describing the relationship between Cav1, ERK, Snail, SMAD2/3, SMAD1-5-8 and EMT master genes, so we defend the usefulness of this simplified model as a first pass. Interestingly, this model indicates (based on our findings) that ERK can simultaneously activate SMAD2-3 while inhibiting SMAD1-5.

We have taken the Reviewer's criticisms as an opportunity to improve our model. We realized that the model did not contain the word "EMT", whose link with Cav1 in a non-tumor context and in this direction (opposite to the one often found in cancer) is novel. Moreover, based on data obtained in our proteomics analysis, we also add proteins related to TGF-beta1 activation. The new model is shown as new **Fig. 9**.

Overall, this study needs to be much better documented and should attempt to provide more than a phenomenological description of EMT and address, in greater depth, the role of caveolae with respect to TGF signaling.

As reported in the Discussion, mechanistic models of the role of *Cav1* (different from the role played by the structural feature, *caveolae*) in TGF-beta signaling have already been provided by Mike Lisanti and G.M. di Guglielmo (Di Guglielmo et al, 2003; Razani et al, 2001). Rob Parton´s group recently published an interesting study on the effect of Cav1 in Ras nanocluster reorganization (Ariotti et al, 2014) that perfectly fits with our discoveries. Our goal in the present study was rather to expand, deepen and challenge concepts previously known from cell biology

studies into an 'interesting medical problem', by focusing on 'the interface between clinical research and basic biology', which fits the scope EMBO Molecular Medicine.

Minor remarks
Referencing should be proof-read.
Snail should be changed to Snail-1 throughout.
Changes have been made according to the Reviewer's suggestions.

REFERENCES

Ariotti N, Fernandez-Rojo MA, Zhou Y, Hill MM, Rodkey TL, Inder KL, Tanner LB, Wenk MR, Hancock JF, Parton RG (2014) Caveolae regulate the nanoscale organization of the plasma membrane to remotely control Ras signaling. *The Journal of cell biology* **204:** 777-792

Aroeira LS, Aguilera A, Sanchez-Tomero JA, Bajo MA, del Peso G, Jimenez-Heffernan JA, Selgas R, Lopez-Cabrera M (2007) Epithelial to mesenchymal transition and peritoneal membrane failure in peritoneal dialysis patients: pathologic significance and potential therapeutic interventions. *J Am Soc Nephrol* **18:** 2004-2013

Di Guglielmo GM, Le Roy C, Goodfellow AF, Wrana JL (2003) Distinct endocytic pathways regulate TGF-beta receptor signalling and turnover. *Nature cell biology* **5:** 410-421

Echarri A, Muriel O, Pavon DM, Azegrouz H, Escolar F, Terron MC, Sanchez-Cabo F, Martinez F, Montoya MC, Llorca O, Del Pozo MA (2012) Caveolar domain organization and trafficking is regulated by Abl kinases and mDia1. *J Cell Sci* **125:** 3097-3113

Hollestelle A, Peeters JK, Smid M, Timmermans M, Verhoog LC, Westenend PJ, Heine AA, Chan A, Sieuwerts AM, Wiemer EA, Klijn JG, van der Spek PJ, Foekens JA, Schutte M, den Bakker MA, Martens JW (2013) Loss of E-cadherin is not a necessity for epithelial to mesenchymal transition in human breast cancer. *Breast cancer research and treatment* **138:** 47-57

Lu Z, Ghosh S, Wang Z, Hunter T (2003) Downregulation of caveolin-1 function by EGF leads to the loss of E-cadherin, increased transcriptional activity of beta-catenin, and enhanced tumor cell invasion. *Cancer Cell* **4:** 499-515

Muriel O, Echarri A, Hellriegel C, Pavon DM, Beccari L, Del Pozo MA (2011) Phosphorylated filamin A regulates actin-linked caveolae dynamics. *J Cell Sci* **124:** 2763-2776

Pinilla SM, Honrado E, Hardisson D, Benitez J, Palacios J (2006) Caveolin-1 expression is associated with a basal-like phenotype in sporadic and hereditary breast cancer. *Breast cancer research and treatment* **99:** 85-90

Razani B, Zhang XL, Bitzer M, von Gersdorff G, Bottinger EP, Lisanti MP (2001) Caveolin-1 regulates transforming growth factor (TGF)-beta/SMAD signaling through an interaction with the TGF-beta type I receptor. *J Biol Chem* **276:** 6727-6738

Richter T, Floetenmeyer M, Ferguson C, Galea J, Goh J, Lindsay MR, Morgan GP, Marsh BJ, Parton RG (2008) High-resolution 3D quantitative analysis of caveolar ultrastructure and caveola-cytoskeleton interactions. *Traffic* **9:** 893-909

Rothberg KG, Heuser JE, Donzell WC, Ying YS, Glenney JR, Anderson RG (1992) Caveolin, a protein component of caveolae membrane coats. *Cell* **68:** 673-682

Salem AF, Bonuccelli G, Bevilacqua G, Arafat H, Pestell RG, Sotgia F, Lisanti MP (2011) Caveolin-1 promotes pancreatic cancer cell differentiation and restores membranous E-cadherin via suppression of the epithelial-mesenchymal transition. *Cell Cycle* **10:** 3692-3700

Strippoli R, Benedicto I, Perez Lozano ML, Pellinen T, Sandoval P, Lopez-Cabrera M, del Pozo MA (2012) Inhibition of transforming growth factor-activated kinase 1 (TAK1) blocks and reverses epithelial to mesenchymal transition of mesothelial cells. *PloS one* **7:** e31492

Figure R1

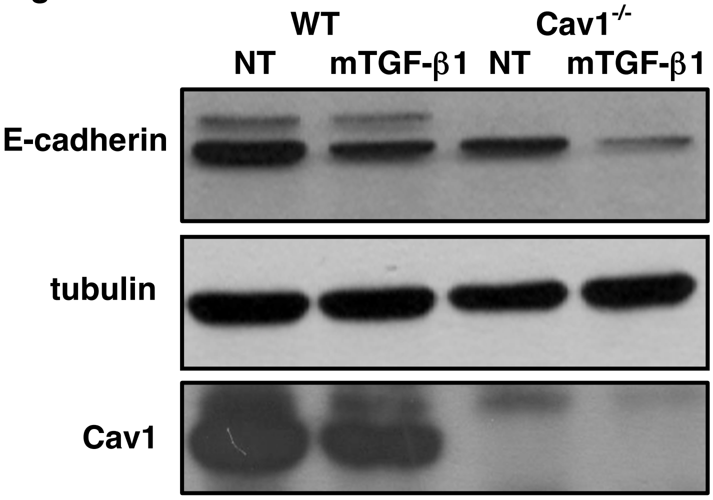

Figure R1 E-cadherin downregulation upon stimulation with murine TGF-b1. Murine MCs were stimulated for 48 h with murine TGF-b1 (2ng/ml). E-cadherin expression was analyzed by WB from cell lysates.

Figure R2: Magnification of Figure 7F

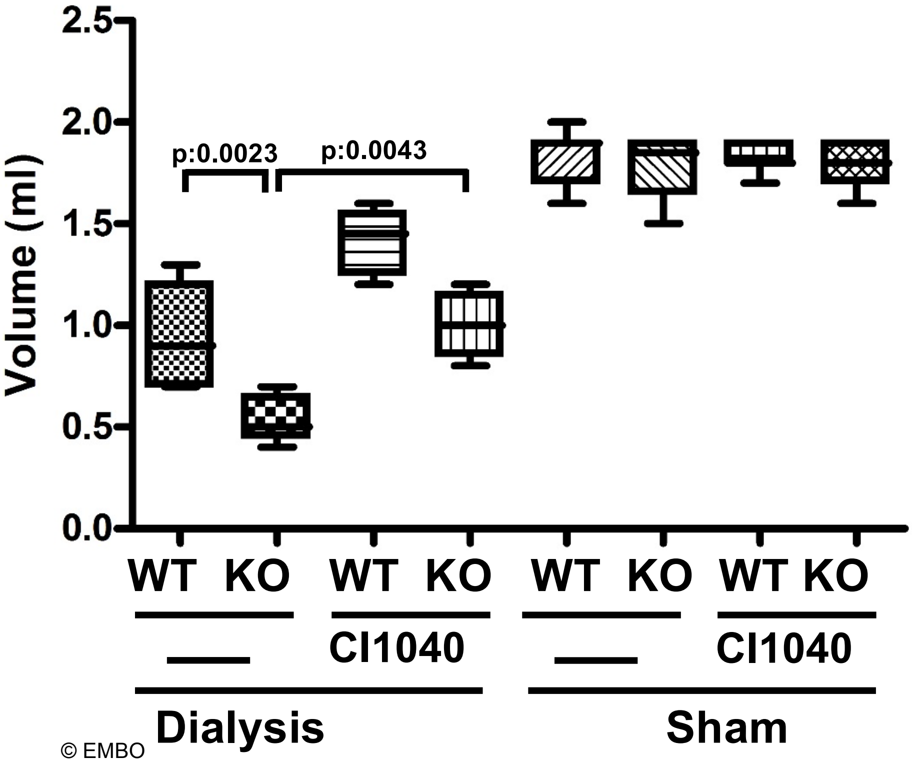

5th Editorial Decision 11November 2014

Thank you for the submission of your revised manuscript to EMBO Molecular Medicine. We have now received the enclosed reports from the reviewers that were asked to re-assess it. As you will see, while Reviewer 3 is globally supportive, Reviewer 2 remains negagtive with respect to suitability of your manuscript for our title. Nevertheless, considering that s/he does not question the quality and integrity of your data and experimentation, I am pleased to inform you that we will be able to accept your manuscript pending the following final amendments (please consider all items carefully):

1) As per our Author Guidelines, the description of all reported data that includes statistical testing must state the name of the statistical test used to generate error bars and P values, the number (n) of independent experiments underlying each data point (not replicate measures of one sample), and the actual P value for each test (not merely 'significant' or 'P < 0.05').

2) I note that the image quality of the tubulin blots in Fig. D blots is not ideal (especially LCav1 lanes). The resolution appears low and the bands appear blocky/blurry when magnifying. Please use better images as these issues could lead to problems when the production team tries to resize these images for the final manuscript.

3) Although I note that your animal experiments are carefully described, the manuscript must include a statement in the Materials and Methods identifying the institutional and/or licensing committee approving the experiments, including relevant details (some of which you provide) such as how many animals were used, of which gender, at what age, which strains, if genetically modified, on which background, housing details, etc. In general, we encourage authors to follow the ARRIVE guidelines for reporting studies involving animals. Please see the EQUATOR website for details: http://www.equator-network.org/reporting-guidelines/improving-bioscience-research-reporting-the-arrive-guidelines-for-reporting-animal-research/

4) We are now encouraging the publication of source data, particularly for electrophoretic gels and blots, with the aim of making primary data more accessible and transparent to the reader. Would you be willing to provide a PDF file per figure that contains the original, uncropped and unprocessed scans of all or at least the key gels used in the manuscript? The PDF files should be labeled with the appropriate figure/panel number, and should have molecular weight markers; further annotation may be useful but is not essential. The PDF files will be published online with the article as supplementary "Source Data" files. If you have any questions regarding this just contact me.

5) Every published paper now includes a 'Synopsis' to further enhance discoverability. Synopses are displayed on the journal webpage and are freely accessible to all readers. They include a short standfirst (to be written by the editor) as well as 2-5 one sentence bullet points that summarise the paper (to be written by the author). Please provide the short list of bullet points that summarise the key NEW findings. The bullet points should be designed to be complementary to the abstract - i.e. not repeat the same text. We encourage inclusion of key acronyms and quantitative information. Please use the passive voice. Please attach these in a separate file or send them by email, we will incorporate them accordingly.

6) May I suggest inclusion of peritoneal dialysis in the title?

Please submit your revised manuscript within two weeks. I look forward to seeing a revised form of your manuscript as soon as possible.

***** Reviewer's comments *****

Referee #2 (Remarks):

Reviewer 2 remains unconvinced with respect to the novelty and impact of the findings. Reviewer 2

does not question the scientific integrity of the observations, nor the quality of the data presented. reviewer 2 simply thinks that the results reported are more suitable for a more specialized journal, and nothing in the revised version has fundamentally changed with respect to this assessment.

Referee #3 (Comments on Novelty/Model System):

Peritoneal dialysis is still practiced today as an ultimate method to bypass renal dialysis in end-stage nephropathies

Referee #3 (Remarks):

The authors have made much effort to respond to the reviewer's criticisms. The phenotype of mesothelial cells is much better documented. The manuscript quality is significantly improved
